# Pharmacological Properties, Molecular Mechanisms, and Pharmaceutical Development of Asiatic Acid: A Pentacyclic Triterpenoid of Therapeutic Promise

**DOI:** 10.3389/fphar.2018.00892

**Published:** 2018-09-04

**Authors:** Mohamed Fizur Nagoor Meeran, Sameer N. Goyal, Kapil Suchal, Charu Sharma, Chandragouda R. Patil, Shreesh K. Ojha

**Affiliations:** ^1^Department of Pharmacology and Therapeutics, College of Medicine and Health Sciences, United Arab Emirates University, Al Ain, United Arab Emirates; ^2^SVKM's Institute of Pharmacy, Dhule, India; ^3^Department of Pharmacology, R. C. Patel Institute of Pharmaceutical Education and Research, Shirpur, India; ^4^Department of Internal Meicine, College of Medicine and Health Sciences, United Arab Emirates University, Al Ain, United Arab Emirates

**Keywords:** asiatic acid, pharmacological properties, phytochemicals, triterpenoids, small molecule, *C. asiatica*

## Abstract

Asiatic acid (AA) is a naturally occurring aglycone of ursane type pentacyclic triterpenoids. It is abundantly present in many edible and medicinal plants including *Centella asiatica* that is a reputed herb in many traditional medicine formulations for wound healing and neuropsychiatric diseases. AA possesses numerous pharmacological activities such as antioxidant and anti-inflammatory and regulates apoptosis that attributes its therapeutic effects in numerous diseases. AA showed potent antihypertensive, nootropic, neuroprotective, cardioprotective, antimicrobial, and antitumor activities in preclinical studies. In various *in vitro* and *in vivo* studies, AA found to affect many enzymes, receptors, growth factors, transcription factors, apoptotic proteins, and cell signaling cascades. This review aims to represent the available reports on therapeutic potential and the underlying pharmacological and molecular mechanisms of AA. The review also also discusses the challenges and prospects on the pharmaceutical development of AA such as pharmacokinetics, physicochemical properties, analysis and structural modifications, and drug delivery. AA showed favorable pharmacokinetics and found bioavailable following oral or interaperitoneal administration. The studies demonstrate the polypharmacological properties, therapeutic potential and molecular mechanisms of AA in numerous diseases. Taken together the evidences from available studies, AA appears one of the important multitargeted polypharmacological agents of natural origin for further pharmaceutical development and clinical application. Provided the favorable pharmacokinetics, safety, and efficacy, AA can be a promising agent or adjuvant along with currently used modern medicines with a pharmacological basis of its use in therapeutics.

## Introduction

The naturally occurring plants and plant-derived non-nutritive compounds termed phytochemicals received attention for their possible utilization in drug discovery and development. These natural compounds often used in dietary/nutritional intervention or as a template for drug discovery are gaining popularity for pharmacological evaluations due to potential efficacy and safety in numerous diseases (Sharma et al., 2016). Among many phytochemicals, triterpenoids belong to one of the major classes of phytochemicals with over 20000 members isolated and recognized, until date. In the past few years, numerous triterpenoids have shown beneficial in the experimental studies and believed to contribute to health promoting properties of edible plants including fruits, vegetables, and spices (Yan et al., 2014b).

In triterpenoids, Asiatic acid (AA), a pentacyclic triterpenoid has gained enormous attention due to its polypharmacological properties and therapeutic potential in numerous diseases (James and Dubery, 2009; Kamble et al., 2014, 2017). AA found in many edible and ornamental, which are popular in traditional medicines and contribute to their thrapeutic benefits (James and Dubery, 2009; Yin, 2015). AA showed to modulate many enzymes, receptors, growth factors, transcription factors, apoptotic proteins, and cell signaling cascades, which attributes pharmacological effects (Kamble et al., 2014, 2017; Patil et al., 2015). Until now, about 250 reports are available which demonstrate promising therapeutic indications, pharmacological effects, pharmacokinetic properties, physicochemical properties, and molecular mechanisms underlying the therapeutic benefits of AA. In experimental studies, AA showed numerous pharmacological activities such as antioxidant, anti-inflammatory, hepatoprotective, cardioprotective, neuroprotective, gastroprotective, and anticancer properties.

The present review aims to represent the available scientific reports on therapeutic potential and underlying pharmacological and molecular mechanisms of AA. The review also discusses the challenges and prospects on the pharmaceutical and clinical development of AA including pharmacokinetics, physicochemical properties, drug delivery, analysis, and structural modifications and drug delivery approaches. AA, chemically an aglycone of ursane type pentacyclic triterpenoid is the chief bioactive constituent from the extract of tropical medicinal plant *Centella asiatica* L. (*C. asiatica*, family, Umbelliferae). This plant is indigenous to Africa, Oceanic countries, and Southeast Asian countries including Indian subcontinent. It is widely consumed in diets as salads, vegetable and in drinks as nutraceutical preparations. Since 3000 years, this plant is acclaimed for its medicinal properties in Chinese traditional and Indian Ayurvedic medicine to promote general health (Nasir et al., 2011, 2012; Zhang et al., 2012). Traditionally, it is indicated for use in wound healing in the pharmacopeia of numerous countries including India, China, Germany, and European countries (James and Dubery, 2009; Thong-On et al., 2014). Though, *C. asiatica* is enlisted in the category of endangered and threatened medicinal plants due to lack of its organized cultivation and over-exploitation of wild resources by the International Union for Conservation of Nature and Natural Resources (IUCN) and Technology Information, Forecasting and Assessment Council (TIFAC) of the Department of Biotechnology, India (Singh et al., 2010).

AA has been shown useful in wound healing, liver fibrosis, cerebral ischemia, dementia, hyperglycaemia, metabolic syndrome, obesity, Alzheimer's, and Parkinson's diseases. The plants containing AA are in use in traditional and folk medicine for beneficial role in many diseases such as depression, memory, stress, wound healing, heart diseases, and cancer. These herbal preparations are available as ointment, dentifrice and cosmetic for dermal disorders, wound healing, venous insufficiency, and microangiopathy (Kim et al., 2009). The extract formulation of *C. asiatica* is available in the name of ECa 233 containing about 80% triterpenoid glycosides such as madecassoside (53.1%) and asiaticoside (32.3%) and madecassol containing triterpenes such as AA, madecisic acid and asiaticoside (Anukunwithaya et al., 2017). Another titrated formulation of *C. asiatica* contains three terpenes viz. AA (30%), madecassic acid (30%), and asiaticoside (40%) and popularly used for wound healing actions (Bylka et al., 2014).

## Sources, chemistry, and physicochemical properties of AA

Until now, AA has been characterized in more than fifty plant species as enlisted in Table 1. In plants, AA is is biosynthesized in by cyclization of squalene and abundantly present in the leaves, flowers and aerial parts with traces in bark, stem, roots, and rhizomes. The extraction of the bioactive compounds from plants is critical to establish standardization and quality control in pharmaceutical and chemical industry along with ensuring safety, efficacy of the products for human use. Gaining improved yield in less time and minimum consumption of organic solvents are the challenges in extraction of the plants. The extractions of AA from plant extracts performed using methanol, ethanol, hexane, water, and ethyl acetate etc. AA also extracted from *C. asiatica* using extraction solvent; subcritical water that provided higher extraction yields than traditional liquid solvent extraction with methanol or ethanol at room temperature (Kim et al., 2009). Supercritical fluid extraction emerges as a potential alternative to conventional liquid solvent extractions due to low extraction yields, long extraction times, and residual toxic organic solvents in final products (Reverchon and De Marco, 2006).

**Table 1:** The plants wherein asiatic acid recognized as a major bioactive constituent.

**S. No**.	**Name of plants**	**Plant parts**	**References**
1	*Actinida arguta*	Roots	Jang et al., 2008
2	*Actinida macrosperma*	Roots	Ding et al., 2018
3	*Akebia trifoliata*	Stem	Gao et al., 2009
4	*Averrhoa carambola*	Whole plant	Yin, 2015
5	*Brassica juncea*	Whole plant	Yin et al., 2012
6	*Centella asiatica*	Leaves	Alqahtani et al., 2015
7	*Citrus microcarpa Bonge*	Whole plant	Yin, 2015
8	*Cichorium intybus L*.	Whole plant	Street et al., 2013
9	*Clematoclethra scandens*	Whole plant	Xiao et al., 2013
10	*Combretum laxum*	Stems	Bisoli et al., 2008
11	*Combretum nelsonii*	Leaves	Masoko et al., 2008
12	*Combretum fragrans i*	Leaves	Dawé et al., 2018
13	*Glechoma hederacea L*.	Whole plant	Yin, 2015
14	*Eucommiae Cortex*	Whole plant	Yan et al., 2018
15	*Gynura bicolor*	Whole plant	Yin, 2015
16	*Hemerocallis fulva*	Whole plant	Yin, 2015
17	*Ilex cornuta*	Leaves	Yao et al., 2009
18	*Ilex pubescens*	Roots	Jiang et al., 2013
19	*Lagerstroemia speciosa*	Leaves	Hou et al., 2009
20	*Ligustrum lucidum Ait*.	Whole plant	Wang, 2014
21	*Lonicera macranthoides*	Roots	Liu et al., 2014
22	*Maytenus procumbens*	Leaves	Momtaz et al., 2013
23	*Melastoma dodecandrum*	Leaves	Yan et al., 2014a
24	*Melastoma malabathricum*	Leaves	Wong et al., 2012
25	*Mucuna birdwoodiana*	Stalk	Ding et al., 1991
26	*Ocimum basilicum*	Whole plant	Yin et al., 2012
27	*Oenothera cheiranthifolia*	Whole plant	Nakanishi et al., 2007
28	*Oenothera laciniata*	Whole plant	Yoon et al., 2009
29	*Potentilla chinesis*	Whole plant	Wei et al., 2013
30	*Psiloxylon mauritianum Baillon*	Whole plant	Mahomoodally et al., 2013
31	*Psidium guajava*	Leaves	Begum et al., 2002
32	*Punica granatum L*.	Seed	Wu and Tian, 2017
33	*Sargentodoxa cuneata*	Stems	Li et al., 2015
34	*Salvia miltiorrhiza Bunge*	Roots	Tung et al., 2017
35	*Schefflera octophylla Harms*	Bark	Chen et al., 2015
36	*Shorea robusta*	Oleoresin	Bharitkar et al., 2013
37	*Schisandra grandiflora*	Stem	Shi et al., 2016
38	*Symplocos lancifolia*	Leaves	Acebey-Castellon et al., 2011
39	*Szygium guineense*	Leaves	Djoukeng et al., 2005
40	*Terminalia arjuna*	Fruit	Hussain et al., 2014
41	*Terminalia catappa L*.	Leaves	Gao et al., 2004
42	*Ugni molinae*	Leaves	Aguirre et al., 2006
43	*Weigela subsessilis*	Leaves and stems	Thuong et al., 2005

Due to its abundant presence in many plants, AA used as a main biomarker component in numerous plant extract based formulations for standardization and quality assurance (Lee et al., 2014). The contents of AA in numerous plant species cultivated, harvested, and habituated from diverse geographic and biodiversity regions have been studied using modern bioanalytical instrumentation techniques. The time of harvesting affects the amount of major triterpenoids and phenolic compounds in *C. asiatica* collected from a particular area at different times and months. In Australia, it was found that harvesting *C. asiatica* during summer seasons yields higher amount of triterpenoids including AA (Alqahtani et al., 2015). Puttarak and Panichayupakaranant (2012) has revealed that the leaves of *C. asiatica* contain the highest amount of triterpenoids with a total amount of 19.5 mg/g. The amount of triterpenoids found varying with the place of cultivation and harvestation period. *C. asiatica* plants harvested in the Trang province of Thailand during March provides the higher amount of total pentacyclic triterpenes (37.2 mg/g dry powder). The plants from Songkhla province of Thailand provides highest amount (37.4 mg/g dry powder) when harvested in December. Whereas, *C. asiatica* collected from Nakornsrithammarat and Ratchaburi, Thailand gave the lowest content of total pentacyclic triterpenoids across all harvesting periods.

**Figure 1:**
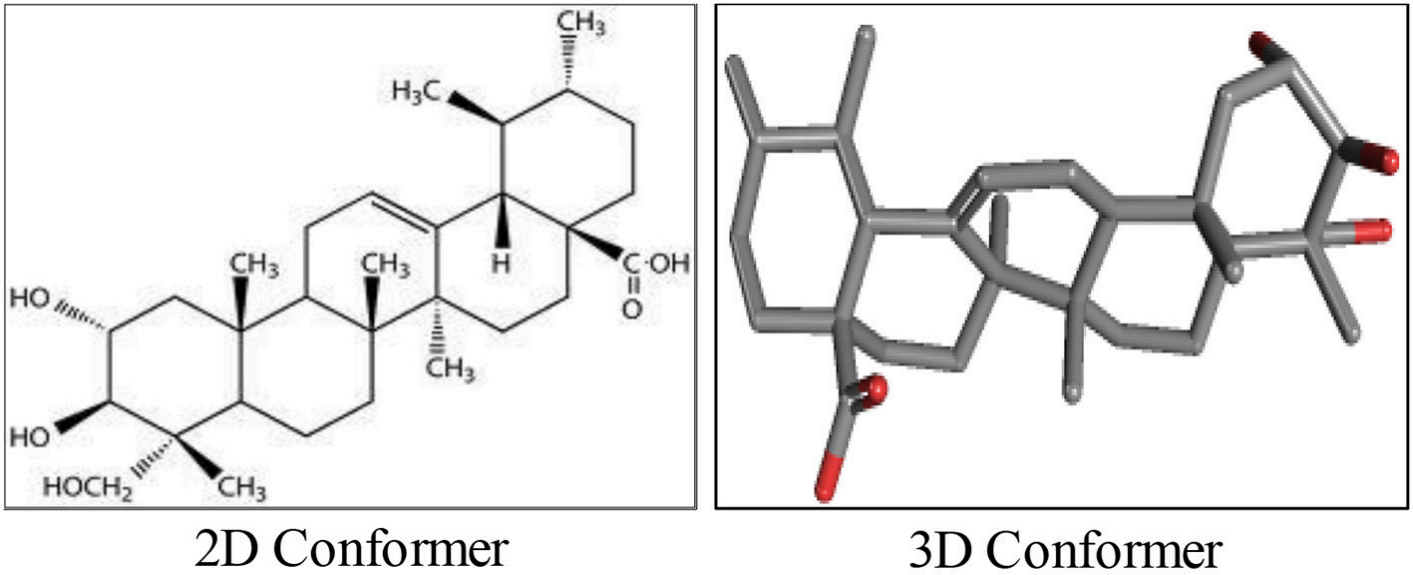
The chemical structure of asiatic acid.

AA, chemically known as [trans-(1R, 9S)-8-Methylene-4, 11, 11-trimethylbicyclo [7.2.0] undec-ene] is also recognized by several other synonyms or names as available in the NCBI compound library based on the contributions by numerous investigators, researchers and organizations. The synonyms and physical properties of AA are represented in Table 2. The 2D structure and 3D conformers are represented in Figure 1. The chemical descriptors and physicochemical properties of AA are represented in Table 3. AA is poorly soluble or miscible in water. It is stable in saline and dissolves at the concentration of 0.1583 mg/mL in saturated saline (Yuan et al., 2015). AA undergoes rapid metabolism that makes it less bioavailable. Asiaticoside, a major component present in the extract of *C. asiatica* also converted to AA following the hydrolytic degradation of the sugar moiety (Rush et al., 1993). Rafat et al. (2008) investigated the role of physicochemical factors such as solubility, lipohilicity, critical micelle concentrations (CMC), and surface tension to micellization and solution properties of AA. The CMC and surface tension of AA were 15 ± 2 M and 64.1 mN/m, respectively. The aggregation numbers and molecular association were between 5 and 7 molecules in solution 5 to 7.

**Table 2:** Physical properties and synonyms of asiatic acid.

Solubility	In water, 5.98X10-2 mg/L at 25 deg C (est) US EPA; Estimation Program Interface (EPI) Suite. Ver.3.12. Nov 30, 2004. Available from, as of Oct 27, 2008: http://www.epa.gov/oppt/exposure/pubs/episuitedl.htm
Vapor pressure	1.17X10-17 mm Hg at 25 deg C (est) US EPA; Estimation Program Interface (EPI) Suite. Ver.3.12. Nov 30, 2004. Available from, as of Oct 27, 2008: http://www.epa.gov/oppt/exposure/pubs/episuitedl.htm
LogP	log Kow = 5.32 (est) US EPA; Estimation Program Interface (EPI) Suite. Ver.3.12. Nov 30, 2004. Available from, as of Oct 27, 2008: http://www.epa.gov/oppt/exposure/pubs/episuitedl.htm
Dissociation constants	pKa = 4.7 (est) SPARC; pKa/property server. Ver 3. Jan, 2006. Available from as of Oct 27, 2008: http://ibmlc2.chem.uga.edu/sparc/
Synonyms of asiatic acid	Asiatic acid, Dammarolic acid, NSC 166063, CHEBI:2873, CHEMBL404313, (2|A,3|A)-2,3,23-trihydroxyurs-12-en-28-oic acid, Asiantic acid, (1S,2R,4aS,6aR,6aS,6bR,8aR,9R,10R,11R,12aR,14bS)-10,11-dihydroxy-9-(hydroxymethyl)-1,2,6a,6b,9,12a-hexamethyl-2,3,4,5,6,6a,7,8,8a,10,11,12,13,14b-tetradecahydro-1H-picene-4a-carboxylic acid, 2alpha,23-Dihydroxyursolic acid, (2alpha,3beta)-2,3,23-trihydroxyurs-12-en-28-oic acid
IUPAC name	(1S,2R,4aS,6aR,6aS,6bR,8aR,9R,10R,11R,12aR,14bS)-10,11-dihydroxy-9-(hydroxymethyl)-1,2,6a,6b,9,12a-hexamethyl-2,3,4,5,6,6a,7,8,8a,10,11,12,13,14b-tetradecahydro-1H-picene-4a-carboxylic acid - (from PubChem)
InChI	Computed International Chemical Identifier (InChI) from the chemical structure using the International Union of Pure and Applied Chemistry (IUPAC) standard. InChI = 1S/C30H48O5/c1-17-9-12-30(25(34)35)14-13-28(5)19(23(30)18(17)2)7-8-22-26(3)15-20(32)24(33)27(4,16-31)21(26)10-11-29(22,28)6/h7,17-18,20-24,31-33H,8-16H2,1-6H3,(H,34,35)/t17–,18+,20–,21–,22–,23+,24+,26+,27+,28–,29–,30+/m1/s1—(from PubChem)
InChI key	Computed International Chemical Identifier hash (InChIKey) from the chemical structure using the International Union of Pure and Applied Chemistry (IUPAC) standard. JXSVIVRDWWRQRT-UYDOISQJSA-N - (from PubChem)
Canonical SMILES	Computed Simplified Molecular-Input Line-Entry System (SMILES) from the chemical structure devoid of isotopic and stereochemical information. CC1CCC2(CCC3(C( = CCC4C3(CCC5C4(CC(C(C5(C)CO)O)O)C)C)C2C1C)C)C( = O)O - (from PubChem)
Isomeric SMILES	Computed Simplified Molecular-Input Line-Entry System (SMILES) from the chemical structure containing isotopic and stereochemical information. SMILES written with isotopic and chiral specifications are collectively known as “isomeric SMILES.” C[C@@H]1CC[C@@]2(CC[C@@]3(C( = CC[C@H]4[C@]3(CC[C@@H]5[C@@]4(C[C@H]([C@@H]([C@@]5(C)CO)O)O)C)C)[C@@H]2[C@H]1C)C)C( = O)O-(from PubChem)
[ CAS	464-92-6—(from European Chemicals Agency—ECHA, ChemIDplus)
EC number:	482-720-9- (from European Chemicals Agency—ECHA)
UNII 9PA5A687X5	610-307-7—(from European Chemicals Agency—ECHA) (from FDA/SPL Indexing Data)

*This list is compiled from the pubmed database*.

**Table 3:** The physicochemical properties of asiatic acid.

**Molecular formula**	**C_30_H_48_O_5_**
Molecular weight	488.709 g/mol
XLogP	5.7
Hydrogen bond donor count	4
Hydrogen bond acceptor count	5
Rotatable bond count	2
Exact mass	488.35 g/mol
Monoisotopic mass	488.35 g/mol
Topological polar surface area	98 A^2^
Heavy atom count	35
Formal charge	0
Complexity	930
Isotope atom count	0
Defined atom stereocenter count	12
Undefined atom stereocenter count	0
Defined bond stereocenter count	0
Undefined bond stereocenter count	0
Covalently-bonded unit count	1

## Synthesis of asiatic acid and its derivatives

Asiaticoside, another triterpenoid constituent of *C. asiatica* has been shown to provide AA upon saponification as well as hydrolysis. Structurally, AA at C-2, C-3, and C-23 positions possesses three hydroxyl groups, an olefin group at C-12 position and a carboxylic acid group at C-28 position. Many investigators to synthesize semisynthetic derivatives following structural modifications using the structure-activity relationships and combinatorial chemistry often utilize these functional groups. Numerous bioactive AA derivatives were synthesized by modifications at C-11 and C-28 positions. The modified derivatives appear more potent, have a higher bioavailability and exhibit improved activity against key signaling pathways regulating inflammation. Based on numerous findings, the derivatives were found to be more potent with optimal efficacy and minimal toxicity. Till date, a large number of derivatives of AA have been synthesized and their structures were confirmed using analytical instrumentation such as infrared spectroscopy, Proton Nuclear magnetic resonance Spectroscopy, High Resolution Mass Spectrometry and Carbon 13 Nuclear magnetic resonance Spectroscopy.

## Pharmacokinetic properties of asiatic acid

The determination of pharmacokinetic data is a vital step in clinical drug development. It begins with estimating first dose size in healthy volunteers along with an optimal route of administration to achieve desired characteristics for development including reasonable systemic bioavailability. In previous years, it has been observed that majority of nature derived small molecules could not progress to clinical studies due to poor pharmacokinetic properties. It is a well-known fact that pharmacokinetics and bioavailability constitute major barriers in drug development. They account for about 16% of failures of molecules in Phase I trials in 605 terminated candidates for drug development by major pharmaceutical companies (Waring et al., 2015). Although accruing data from numerous experimental studies demonstrated the pharmacological effects and therapeutic benefits of AA against many diseases, but non-availability of pharmacokinetic data was the major factor limiting its clinical use. In modern medicine, randomized clinical trials are vital steps in establishing the safety and efficacy of AA as a potential agent for use in therapeutics in numerous diseases. Not only AA, but also many natural molecules such as resveratrol, curcumin, epigallocatechin gallate, and baicalein found promising drug candidates in preclinical models but due to their limited bioavailability and physicochemical properties, clinical development, and usage for therapeutic benefits were limited. The available preclinical data on AA positively suggest a promising future for clinical studies.

Numerous studies have characterized the pharmacokinetics of AA in rats and dogs (Chassaud et al., 1971; Grimaldi et al., 1990; Zheng and Wang, 2009; Pan et al., 2010; Nair et al., 2012; Yin et al., 2012; Yuan et al., 2015). For the first time, Chassaud et al. (1971) investigated the metabolism of AA in rats. Recently, Xia et al. (2015) identified 10 metabolites of AA that were formed mainly by hydroxylation, dehydrogenation, or dehydroxylation reactions following Phase I metabolism. AA appears to be a potent inhibitor of CYP2C9 (Ki = 9.1 mg/ml) isoform of P450 enzymes. The potent inhibitory effect of AA on CYP2C9 showed its potential to cause drug-herb interactions especially for the drugs metabolized by this isoform (Pan et al., 2010). Zheng and Wang (2009) determined pharmacokinetics of orally administered AA in beagle dogs. AA showed a *T*_1/2_ of 4.29 h; *T*_max_, 2.70 h; *C*_max_, 0.74 μg/mL; *AUC*_0–*t*_ and *AUC*_0−∞_, 3.74 and 3.82 μg h/mL, respectively. Yuan et al. (2015) developed a high performance liquid chromatography (HPLC) method and studied the pharmacokinetics of AA administered orally (20 mg/kg) or intravenously (2 mg/kg) in rats and *in vitro* in Caco-2 cells. Following oral ingestion, AA showed the following PK parameters: *T*_*max*_ (0.5 h), *C*_max_ (0.394ng mL^−1^), *t*_1/2_ (0.642 h), *AUC*_(0–24)_ (0.702ng mL^−1^ h^2^), *AUC*_(0−∞)_ (0.766ng mL^−1^ h^2^), *AUMC*_(0–24)_ (1.213ng mL^−1^ h^2^), *AUMC*_(0−∞)_ (1.641ng mL^−1^ h^2^), *CL* (6.682 L h ^−1^), *MRT* (0.668 h), and bioavailability (16.25%). Upon intravenous injection, AA showed *T*_max_ (0.08 h), *C*_max_ (1.176ng mL^−1^), *t*_1/2_ (0.348 h), *AUC*_(0–24)_ (0.432ng mL^−1^ h^2^), *AUC*_(0−∞)_ (0.482ng mL^−1^ h^2^), *AUMC*_(0–24)_ (0.109ng mL^−1^ h^2^), *AUMC*_(0−∞)_ (0.186ng mL^−1^ h^2^), *CL* (4.186 L h ^−1^), and *MRT* (0.258 h). AA after oral dosing achieves a maximum plasma concentration after 30 min that demonstrates its rapid absorption in the blood supply though it is absorbed poorly and follows passive diffusion with a major site of absorption in jejunum, followed by rapid metabolism in the liver by CYP450 enzymes. Transportation parameters, regional absorption and metabolic rate were studied using Caco-2 cells, rat intestinal perfusion model and rat liver microsomes, respectively. Oral bioavailability is a vital parameter to attain the effective therapeutic levels of the drug and it represents the most optimal route of drug administration. In majority of the preclinical studies, AA was efficacious when administered orally or interaperitoneally. This is noteworthy since oral bioavailability is physiologically and clinically relevant to maximize therapeutic utility.

The lipophilicity, physicochemical properties and availability in brain tissues after administration reasonably supports the neuroprotective potential of AA. Bioavailability in the brain has indicated that AA may cross the blood-brain barrier (BBB) and the attained concentration of AA appear adequate to elicit neuroprotection against neurodegenerative diseases. In regard to drug discovery and development, it is considered that a drug should be able to transport across the BBB if it possesses important physicochemical features such as presence in un-ionized form, partition coefficient (log P) value about 2 or more, molecular weight lesser than 400 DA and cumulative number of hydrogen bonds lesser than 8–10 (Pathan et al., 2009). AA behaves an un-ionized molecule with log *P*-value of 5.7 (highly lipophilic), molecular weight 488.709 g/mol and nine cumulative hydrogen bonds. These properties of AA make it permeable to BBB. However, very little information is available regarding the BBB permeability of AA in the *in vitro* models. However, numerous *in vivo* studies in the neurodegenerative diseases clearly demonstrate the bioavailability of AA in the brain that explicitly demonstrates the neuroprotective effects of AA. However, more *in vivo* pharmacokinetic studies focusing on BBB permeability needed to assess blood-brain cerebrospinal fluid and blood-brain extracellular fluid drug-concentration relationship following variation in drug doses and plasma drug levels. In a study evaluating the neuroprotective potential of AA, following a single intravenous injection to rats at the dose of 10, 25 and 75 mg/kg, the serum concentrations of AA were found to be 2.95, 6.16, and 8.62 μM respectively after 15 min (Lee et al., 2012). The (*C*_max_), *T*_1/2_ and (*AUC*) value of 75 mg/kg AA was 2.00 ± 0.18 h, 9.70 ± 0.82 mg/L, and 27.67 ± 2.91 mg.h/L, respectively. AA was well-tolerated up to a dose of 75 mg/kg (Lee et al., 2012). The bioavailability of AA in the plasma, brain, heart, liver, kidney, colon and bladder is more when fed with AA containing fruits and vegetables for several weeks (Yin et al., 2012; Chao et al., 2016).

The pharmacokinetics of AA present in a total triterpenic fraction of *C. asiatica* was studied in healthy volunteers (Grimaldi et al., 1990). The volunteers received either 30 or 60 mg of single oral doses and after a 7-day treatment either 30 or 60 mg twice-daily oral doses in a randomized crossover design with a 3-week interval between the trails. The time of peak plasma concentration was found unaffected with dosage difference or treatment schemes. However, chronic protocol showed increased half-life, *AUC*_0–24_ and peak plasma concentrations. Rush et al. (1993) described the bioavailability of AA in healthy volunteers, male and female both following administration of equimolar doses of 24 mg of asiaticoside or 12 mg of AA. Orally administered AA exhibited steady state *AUC*_0−12h_ 614 ± 250ng.h/ml compared to 606 ± 316ng.h/ml for asiaticoside. The pharmacological effects and therapeutic benefits of asiaticoside are mediated by *in vivo* metabolic conversion of asiaticoside to AA, the active component of products of *C. asiatica* extract.

The available preclinical and clinical pharmacokinetic data suggest that AA is bioavailable in almost every tissue. It's distributed to many components of the body by binding with albumin (Gokara et al., 2014). Following intravenous injection, asiaticoside gets widely distributed in several organs and is metabolized extensively and recovered as AA in the feces (Hengjumrut et al., 2018). However, for determining first dose size and optimal therapeutic dose, there is an urgent need of studies to optimize the pharmacokinetics in humans, taking support from preclinical efficacy and safety results.

## Pharmaceutical analysis and development of asiatic acid

The triterpenoid ingredients in *C. asiatica* have been identified by thin layer chromatography using mass spectrometry on the silica gel plates following slight modification of the protocol provided in the European Pharmacopeia (Bonfill et al., 2006). The compounds were separated from the extract using a mobile phase consisting of ethyl acetate and methanol and detection with 4-anisaldehyde and further by MALDI-TOF mass spectrometry (Bonfill et al., 2006). The amount of AA in tissue and plasma samples from experimental animals or in pharmaceutical products has been analyzed using HPLC.

Several analytical methods have reported for the determination of AA and validated following the US Food Drug Administration (FDA) guidelines (Morganti et al., 1999; Schaneberg et al., 2003; Rafamantanana et al., 2009; Nair et al., 2012; Yuan et al., 2015). These triterpenes were determined at a wavelength of 220 nm using a gradient system of different solvents mainly water and acetonitrile. In HPLC estimations, novel pre-derivatization method was developed using p-toluidine as a coupling agent to improve sensitivity, as AA possesses a very weak chromophore (Raval et al., 2015). Zheng and Wang (2009) has developed a novel pre-column derivatization RP-HPLC method with UV-Vis detection (248 nm) for quantitative estimation of AA in the plasma.

AA was extracted with n-hexane-dichloromethane-2-propanol (20:10:1, v/v/v) from plasma that has been hydrolysed by acid and derivatized with p-toluidine employing chromatographic separation on C_18_ column using gradient elution in a water-methanol system. The lower limit of quantification [LLOQ] was 0.01 μg/mL in a linear range from 0.01 to 1.5 μg/mL and the extraction recoveries were no less than 65%. The plasma samples found stable for 30 days at −20°C.

## Microbial transformation of asiatic acid

The amount of pentacyclic triterpenoids including AA in different parts (leaves, stolons, petioles, flowers, fruits and nodes with roots) of *C. asiatica* were determined using HPLC (Puttarak and Panichayupakaranant, 2012). Triterpene saponin contains triterpene aglycones (sapogenins) with one or more sugar moieties connected through acetal or ester glycosidic linkages on one or many sites (Yu et al., 2006). Purified recombinant UDP-glucose 28-O-glucosyltransferase was found to exhibit narrow specificity, glucosylating AA at C28 carboxyl, involved in saponin biosynthesis in *C. asiatica* (de Costa et al., 2016). The mRNA of this enzyme was found in the stems, roots, and flowers, with the highest concentration found in the leaves (de Costa et al., 2016). In Thailand, contents of pentacyclic triterpenoids reported to vary according to the harvesting period and geographical localities. In order to enhance triterpene production in *C. asiatica*, manipulation of metabolic pathways showed a promising rise in secondary metabolites (James et al., 2013). Jasmonates plays a critical role in the metabolic pathways of plants for the biosynthesis of secondary metabolites by regulating the expression of genes. In cell suspensions treated with methyl jasmonate, a metabolomic profiling using LC-MS revealed variation in AA, madecassic acid, asiaticoside, and madecassoside as signatory biomarkers and suggested that it could be used to enhance biosynthesis of the targeted centelloids. In order to develop novel derivatives by structural modification of the useful but less available phytoconstituents, the biotransformation strategy has shown an efficient, specific and environment-friendly technology.

Among the available techniques, microbial transformation is vital for natural products. AA upon transformation using endophytic fungus *Umbelopsis isabellina* provides 2α, 3β, 7β, 23-tetrahydroxyurs-12-ene-28-oic acid and 2α,3β,7β,23-tetrahydroxyurs-11-ene-28, 13-lactone (Gao et al., 2015). In another study, AA provides 2α,3β,15α,23-tetrahydroxyurs-12-en-28-oic acid, 2α,3β,21β,23-tetrahydroxyurs-12-en-28-oic acid, 2α,3β,23-trihydroxyurs-12-en-28,30-dioic acid, and 2α,3β,23,30-tetrahydroxyurs-12-en-28-oic acid upon microbial transformation with the strains of *Penicillium lilacinum* (ACCC 31890), *Fusarium equiseti* (CGMCC 3.3658), and *Streptomyces griseus* (CGMCC 4.18). These derivatives also showed cytotoxicity in several cancer cell lines of human origin (Guo F. F. et al., 2012). Huang et al. (2012) demonstrated the capabilities of 25 strains of filamentous fungi; *Fusarium avenaceum* (AS 3.4594) to transform AA that provides 2-oxo-3β,15α,23-trihydroxyurs-12-en-28-oic acid, 3-oxo-2,15α,23-trihydroxyurs-1,12-dien-28-oic acid and 2-oxo-3β,23-dihydroxyurs-12-en-28-oic-acid. Using fungus *Alternaria longipes* (AS 3.2875), AA yields derivatives such as 2α,3β,23,30-tetrahydroxyurs-12-ene-28-oic acid, 2α,3β,22β,23-tetrahydroxyurs-12-ene-28-oic acid and 2α,3β,22β,23,30-pentahydroxyurs-12-ene-28-oic acid (He et al., 2010).

## Drug delivery of asiatic acid

The physicochemical and pharmacokinetic properties of AA including solubility, lipophilicity, absorption, metabolism, elimination rate, and bioavailability are the major barricades in development and delivery of AA as a drug. The current trend in drug discovery and development with natural products are to develop the dosage forms and formulations with improved bioavailability to attain therapeutic effects with favorable pharmacokinetics and negligible adverse effects at the therapeutic doses. Numerous attempts were taken in order to develop formulations containing AA with favorable pharmaceutical characteristics to improve targeted drug delivery options for the treatment of various human diseases and disorders. Several types of formulations such as nanoparticles using albumin, poly glutamic acid or glutathione, transdermal, multiple emulsions, liposomes, solid dispersion complexations were developed to improve drug delivery options for AA. These formulations, upon oral administration found bioavailable in almost every tissue and even upon topical application found bioavailable within the different layers of skin. Therefore, these can be used as a skin permeation enhancing agents that rationalizes the regenerative cosmetic use of extracts containing AA.

A novel formulation Jaluronius CS fluid containing hyaluronic acid 1%, glycerin 5%, and *C. asiatica* stem cells has been recently developed and found to improve skin hydration and skin barrier function for longer duration (Milani and Sparavigna, 2017). A transdermal delivery system containing *C. asiatica* used for the treatment of cellulitis has been evaluated using reversed-phase HPLC coupled with detector photodiode array for the identification of AA (Morganti et al., 1999). Schaneberg et al. (2003) developed an improved qualitative and quantitative HPLC protocol to identify triterpenoids in both methanolic extract and in the preparations of *C. asiatica*. A Phenomenex Aqua 5mu C18 (200 A) column was used as a stationary phase and the gradient mobile phase contained water (0.1% TFA), acetonitrile (0.1% TFA), and methyl tert-butyl ether (0.1% TFA) following detection by UV at 206 nm. In another study, AA along with other triterpenoids was quantified in *C. asiatica* by HPLC-UV and this newly devised method was suggested for routine analysis of AA and samples of *C. asiatica* (Rafamantanana et al., 2009). The method was validated and showed convenience and accuracy for **estimation of AA** in the concentration range of 0.5–2.0 mg/ml with CV <3% for all investigated compounds. The LOD and LOQ of AA were 0.0023 and 0.5 mg/ml respectively.

Shen et al. (2014) also developed a sensitive, specific and reproducible HPLC method for use as a standard in quality control and assurance of *C. asiatica* extracts. In another study, the HPLC fingerprints of *C. asiatica* were developed to establish a good, reliable, reproducible, specific, sensitive and robust method for quality control on samples collected from different parts of China (Lu et al., 2011). Among 15 common peaks in HPLC fingerprints, 5–10 peaks were identified as madecassoside, asiaticoside, quercetin, kaempferol, madecassic acid, and **AA** using reference standards and LC-ESI-MS. Further, Nair et al. (2012) developed US FDA guidelines compliant HPLC/electrospray ionization (ESI)-MS/MS solid-phase extraction method to determine the quantity of AA in the plasma of rats following formation of ammonium adduct of AA separating on a Cosmosil C column employing a gradient flow mobile phase. Colchicine was used as an internal standard. The calibration showed a linear range of 1.02–407.88 ng/mL with 90% mean percentage recovery in samples with optimal precision (intraday and interday) and accuracy. The authors validated the developed method to US FDA guidelines and found it to be sensitive and reproducible to measure AA in the plasma following oral administration of *C. asiatica*.

Xia et al. (2015) has developed LC/IT-MS/MS analytical protocol for rapid screening and identification of the AA metabolites in zebrafish using negative ion mode and collision-induced dissociation to acquire fragmentation pathways of AA. Ten metabolites of AA were formed from phase I metabolism reactions such as hydroxylation, dehydrogenation, and dehydroxylation. AA behaved as a deprotonated molecule [M–H] ^−^ at m/z 487 with 37.57 min of retention time. Following the cleavage of the alicyclic ring, keto-enol tautomerism and Retro-Diels-Alder cleavage on the C ring of AA structures generated the fragmented ions of AA. AS 2-006A, a derivative of AA chemically known as ethoxymethyl 2-oxo-3, 23-O-isopropylideneasiatate was characterized in the rat and human plasma and urine using a mobile phase consisting of acetonitrile: H_2_O (9:1, v/v) that has a flow rate of 1.1 ml/min (Kim et al., 1999). A UV detector set at 205 nm showed the detection limits for AS 2-006A in rat and human plasma were 1 μg/ml, and in rat urine was 2 μg/ml with a retention time of 29.5 min. The lowest interday and intraday coefficient of variation (below 10.8%) was found in the rat's plasma and urine as well as in the plasma of humans. Interday and intraday coefficients of variation of the assay were found to be low (below 10.8%) for the plasma and urine samples of rats as well as in the plasma of human samples. The retention time, detection limits and the intraday and interday coefficients of variation of the assay showed stability, sensitivity and found favorable in regards to pharmaceutical development (Kim et al., 1999). Additionally, the endogenous substances did not cause interferences in this assay.

In another study, Yuan et al. (2015) has developed LC-MS method and found aqueous ammonium acetate an optimal mobile phase for best retention time, response, and intensity for AA in the samples. The method is very specific and sensitive for the determination of pharmacokinetic parameters in the plasma of rats. AA showed LLOD [20.50 ng/mL, signal-to-noise ratio (*S*/*N*)>3] and LLOQ (51.25 ng/mL, *S*/*N*>5) and found stable in the plasma for 8 h at room temperature, 30 days at −40°C, and survive upto 3 freeze–thaw cycles with minimal degradation (*RE* < 15%). Numerous drug delivery designs were developed to improve the solubility, stability and bioavailability of AA. It has been complexed with many polymers including hydroxypropyl-β-cyclodextrin and formulated with Eudragit E100, glycerol, PEG 400, and copovidone. AA is distributed to many compartments of the body by binding with albumin and it has shown bound to the subdomain IIA with hydrophobic and hydrophilic interactions (Gokara et al., 2014). The O/W/O multiple emulsion formulations were prepared to make them stable by protecting from oxidative degradation and provide a modified release upon topical application. The emulsions containing AA and other triterpenes were stable for 6-month storage at room temperature and at 40°C. AA showed percutaneous absorption by stratum corneum, epidermis, and dermis as determined in *in vitro* percutaneous experiment using Franz diffusion cells in nuderats (Laugel et al., 1998).

Pegylated AA loaded nanostructured lipid carriers were developed using solvent diffusion method to promote intestinal absorption of AA following modification with hydrophilic PEG. *In situ* preparation of single pass perfusion model of rat AA showed a favorable and improved absorption kinetics of p-AA-NLC in small intestine (Huang et al., 2016). In Sprague Dawley rats, these nanoformulations showed improved pharmacokinetics and bioavailability as evidenced by arise in *C*_max_ of the drug excretion and elimination half-life *T*_1/2_ with decreased *T*_max_ (Zhang et al., 2017). In drug delivery system, nanoparticles are used to enhance therapeutic efficacy by facilitating solubility and promote targeted drug delivery to the site of action through binding or interacting with the receptors or membranes (Zhou et al., 2018).

Recently, another formulation containing solid lipid nanoparticles of AA tromethamine was developed to evade proteolytic degradation and facilitate sustained release of the drug as well as enhanced bioavailability (Lingling et al., 2016). The formulation was developed using solvent injection method containing lipid component glycerin monostearate (MS) and surfactant component poloxamer 188. The formulation was optimized using Box-Behnken design and dynamic light scattering, scanning electron microscopy, differential scanning colorimetry and X-ray diffraction that determined physicochemical characters and HPLC-MS/MS assessed pharmacokinetics. The formulation showed an average spherical size of 237 nm with a zeta potential of −35.9 mV and EE% of 64.4% with a smooth surface and excellent stability at 4°C. The formulation possesses more bioavailability than AA (about 2.5 times) following a single oral administration in rats. Solid lipid nanoparticles appear to be a promising oral delivery system of AA. In another study, non-drug components such as glyceryl monostearate (MS), glyceryl distearate (DS), and glyceryl tristearate (TS) used in making nanoparticles. These ingredients did not elicit toxicity toward normal SVG P12 cells, whereas the formulation containing AA-loaded MS-SLNs (AA-MS-SLNs) caused selective cytotoxicity of cancer cells (Garanti et al., 2016). AA-MS-SLNs showed a concentration-dependent apoptotic activity on glioma cells and revealed cellular uptake of SLNs by energy-dependent endocytosis that further demonstrate the therapeutic potential of AA-loaded MS-SLNs for the treatment of brain tumors and showed success of AA-MS-SLNs for the pharmaceutical development.

In another approach toward developing a brain-specific drug delivery system, novel bovine serum albumin (BSA) nanoparticles coupled with a natural tripeptide; glutathione was made by desolvation technique. These glutathione-conjugated AA-loaded BSA nanoparticles were intravenously injected into rats at a dose equivalent to 75 mg/kg (Raval et al., 2015). This nanoformulation showed improved bioavailability (10-fold more) than AA in the brain after 5 h. The developed nanoparticle formulation retains AA like a reservoir and reduces the accumulation of free AA in off-target tissues and organs, thus avoids the systemic toxicity of AA.

## Pharmacological and molecular mechanisms of asiatic acid

In recent years, a convincing number of studies have demonstrated the pharmacological and molecular mechanisms of AA in the *in vitro* and *in vivo* studies.

## Modulatory activities of asiatic acid on receptors and enzymes

AA showed to modulate enzymes and receptors that is presented in Tables 4, 5. AA was found to activate PPAR-γ, benzodiazepine site on the GABAA and GABAB receptors. Additionally, it blocks the receptors like angiotensin (AT1), endothelin 1 (ET1), toll-like receptors (TLR-4), and AGE formation. AA inhibits α-glucosidase, leukotriene C4 synthase, glycogen phosphorylases, HMG-CoA-reductases, lipase, prolyl 4-hydroxylase-α/β, β-amyloid formation, AChE, BACE1, eNOS/iNOS, PARP, COX-2, CYP2C9, 2D6, 3A4 enzymes, and promotes induction of matrix metalloproteinase, collagen-1 synthesis, plasminogen-1, β-amyloid clearance, processing, and acetylcholine synthesis.

**Table 4:** The enzyme modulating properties of asiatic acid.

**S. No**.	**Enzyme modulation**	**Experimental model of disease**	**References**
1	α-Glucosidase inhibition	Antidiabetic	Hou et al., 2009
2	Leukotriene C4 synthase pathway	Hepatoprotective	Ma et al., 2009
3	Glycogen phosphorylases inhibition	Diabetes and ischemic diabetic complications	Wen et al., 2008; Zhang et al., 2009
4	HMG Co-reductase inhibition	Anti-hyperlipidemic	Ramachandran et al., 2014
5	Lipase inhibition	Anti-obesity	Jang et al., 2008
6	Prolyl 4-hydroxylase-α/β inhibition	Liver fibrosis	Dong et al., 2004
7	MMP-2 inhibition	UVA-induced Photo aging	Soo Lee et al., 2003
8	β-amyloid inhibition	Alzheimer's disease	Mook-Jung et al., 1999; Jew et al., 2000
10	Plasminogen-1 induction	Keloid	Bian et al., 2013
11	AChE inhibiton	Alzheimer's disease	Nasir et al., 2012
12	β-amyloid clearance and processing	Alzheimer's disease	Patil et al., 2010
13	BACE1 enzyme inhibition	Alzheimer's disease	Patil et al., 2010
14	CYP2C9, 2D6, 3A4 inhibition	Drug-drug interaction	Pan et al., 2010
15	Endothelial and inducible NOS	Metabolic syndrome	Pakdeechote et al., 2014
16	PARP modulation	Dopaminergic neurotoxicity	Park et al., 2005; Tang et al., 2009
17	COX-2 inhibition	Anti-inflammatory	Yoon et al., 2009
18	Acetylcholine synthesis enhancer	Memory impairment in dementia	Kim et al., 2004
19	Lecithinase inhibition	Anti-enterococcal	Wojnicz et al., 2017
20	Myeloperoxidase inhibition	Pancreatitis	Xiao et al., 2017
21	Acetyl CoA carboxylase, uncoupling protein-2 &carnitine palmitoyltransferase-1	Anti-obesity	Rameshreddy et al., 2018
22	Inducible NOS and COX-2 inhibition	Inflammation	Yun et al., 2008
23	P450 (CYP)2E1 inhibitory activity	Alcoholic hepatitis	Wei et al., 2013
24	Inducible NOS and COX-2 inhibition	Anti-inflammatory	Huang et al., 2011
25	Inducible NOS and COX-2 inhibition	Anti-tumorigenesis	Park et al., 2007
26	PARP inhibition	Fulminant hepatitis	Guo W. et al., 2012
27	PARP inhibition	Anti-cancer	Gonçalves et al., 2016
28	MMP-2 and MMP-9 inhibition	Gastric cancer	Jing et al., 2016
29	Janus-activated kinase 2 inhibition	Gastric cancer	Wang et al., 2016
30	Glutamine synthetase activation	Epilepsy	Wang et al., 2018
31	Tyrosine hydroxylase activation	Parkinson's disease	Chao et al., 2016
32	Glycerol-3-phosphate dehydrogenase inhibition	Osteoporosis	Li et al., 2014

**Table 5:** The receptor modulator properties of asiatic acid.

**S. No**.	**Receptor modulation activity**	**Disease model**	**References**
1	PPAR-γ activation	keloid	Bian et al., 2013
2	Angiotensin receptor antagonism	Hypertension	Caballero-George et al., 2004
3	Endothelin-1 receptor antagonism	Hypertension	Caballero-George et al., 2004
4	Toll like receptor-4 inhibition	Alcoholic hepatitis	Wei et al., 2013
5	GABA_B_ receptor agonism	Depression	Nasir et al., 2012
6	GABA_A_ receptor modulation	Anxiety and depression	Ceremuga et al., 2015
7	Receptor of AGE inhibition	Skin aging	Wang, 2014
8	Toll like receptor-4 inhibition	Lung injury	Li and Xiao, 2016
9	Toll-like receptor-2, 4 inhibition	Parkinson's disease	Chao et al., 2016
10	GABAA receptors modulation	Cognition and memory	Hamid et al., 2016
11	PPAR-γ activation	Osteoporosis	Li et al., 2014
12	PPAR-γ activation	Periodontitis	Hao et al., 2017
13	Receptor of AGE inhibition	Brain aging	Chao et al., 2015

## Antioxidant activity of asiatic acid

AA showed to elicit potent antioxidant and free radical scavenging properties involving various pathways. AA produced dose-dependent free radical scavenging activity by countering hydroxyl radicals and superoxide anions. The antioxidant mediated organo-protective effects of AA demonstrated in various experimental models of human diseases. AA is a highly effective chain-breaking antioxidant, which acts against reactive oxygen species (ROS). AA showed to attenuate myeloperoxidase activation and inhibit lipid peroxidation. The inhibitory capacity on lipid peroxidation appears shigher than several well-known antioxidants such as probucol, ascorbic acid, and α-tocopherol. AA also found to augment activities/levels of both enzymatic and non-enzymatic antioxidants.

## Anti-inflammatory activity of asiatic acid

The beneficial effect of AA in inflammatory conditions in several experimental studies showed due to its capacity to regulate pro-inflammatory cytokines and preventing the development and progression of immune-inflammatory disorders. The NF-κB inhibitory activity of AA was further supported by *in silico* and *in vitro* studies (Patil et al., 2015; Kamble et al., 2017). AA ameliorated NF-κB expression in LPS-stimulated RAW264.7 cells, inhibited IKKα/β phosphorylation and interferon-gamma (IFN-γ) activation. Docking studies for the prediction of NF-κB inhibitory activity was carried out using PASS (prediction of activity spectra of substances) software followed by docking of the NEMO/IKKβ association complex (PDB: 3BRV). The compliance was tested with the softened Lipinski's Rule of Five that showed AA has promising potential to be developed as an anti-inflammatory drug against various inflammatory diseases (Patil et al., 2015).

AA isolated from leaves and stems of *Weigela subsessilis* showed anti-complement activity as evidenced by the inhibition of the hemolytic activity of human serum against erythrocytes in micromolar concentrations (Thuong et al., 2006). This activity is attributed to the carboxylic group present in the structure of ursane type triterpenoids. AA also inhibits pathways initiated by the activation of toll-like receptors which leads to the expression of pro-inflammatory cytokines (IL-1β, IL-6, IL-8, and TNF-α) and modulates the immune responses. AA in fluorescent-based assays showed diminished endothelial cell activation on monocyte adhesion in monocytic cell lines (U937) and monocyte migration in HAECs (Fong et al., 2016). The endothelial cell activation plays an important role in the atherogenesis and other chronic inflammatory diseases. AA was shown to inhibit endothelial hyperpermeability, enhanced VCAM-1 expression and improve the levels of soluble CAMs (sE-selectin, sICAM-1, sVCAM-1, and sPECAM-1) provoked by TNF-α. AA neither altered PECAM-1 expression nor inhibited TNF-α-induced increased monocyte adhesion and migration. AA attenuated increased phosphorylation of IκB-α stimulated by TNF-α. AA showed potent immunomodulation due to its inhibitory effect on both Th1/Th2 cytokines that indicates the potential benefits of AA in several autoimmune diseases. Owing to the multimodal anti-inflammatory mechanisms, AA appears an important agent to treat diseases where immune-inflammatory alterations are a common accompaniment in pathogenesis.

## Molecular mechanisms of asiatic acid

AA exhibits multipharmacological properties and multimodal molecular mechanisms in several *in vitro, in vivo*, and *in silico* studies. Although, it is challenging to represent the best molecular target or mechanism, but AA has been reported to modulate many molecular targets by changing their gene expression, signaling pathways, or through direct interaction as summarized in Table 6. AA regulates the expression of cytokines (e.g., TNF-α, IFN-γ, IL-1, IL-4, IL-5, IL-6, IL-10), chemokines (e.g., CINC-1/CXCL1, CXCL1/KC, MIP-2, MCP-1), growth factors (e.g., VEGF, TGF, CTGF, FGF, BDNF), enzymes (e.g., AChE, BChE, NOX, iNOS, FATP4, ACS, CPT1, ACOX, CYPs, COX-2, LOX, MMP9, MPO, NAG, MAPK), signaling molecules (ERK, JNK1/2, PGC-1α, PI3K/Akt, AMPK/CREB, mTOR, Akt), adhesion molecules (e.g., ICAM-1, VCAM-1), apoptosis-related proteins (e.g., Bcl-2, mdm2, cmyb, bax, bak1, Apaf-1, caspases, p53, p38, ATM, DR, Fas), cell cycle proteins, e.g., cyclin D1, g-proteins e.g., Arf6, Rac1, Cdc42, heat shock proteins 60 and P-gP), genes (e.g., *Col1a1, Tgfb1, Timp1, SREBP-1c, SCD1*), and receptors (μ-opioid, PPAR-γ, TLRs). AA also modulates the activity of several transcription factors (e.g., NF-κB, AP-1, SIRT1, STATs) and their signaling pathways. AA showed to cause favorable modulation of IKK/MAPK pathway, ERK, ERK/p38MAPK, Ras/Raf/MEK/ERK, TGF-β1/Smad signaling, IKKβ kinase, TGF-β/Smad3, and NDR1/2 dependent p21WAF1/CIP1 activity. AA inhibited TGF-β1, NF-κB, myeloid differentiation factor-88, complement, Smad3, NLRP3 inflammasome, and induces Smad7 and nerve growth factors. Based on its ability to affect multiple targets, AA has the potential for the prevention and treatment of various diseases.

**Table 6:** The cell signaling pathway modulated by asiatic acid.

**S. No**.	**Modulation of signaling pathway**	**Disease/experimental models**	**References**
1	NF-κB/MAPK (p38, ERK1/2/JNK) pathway	Anti-inflammatory	Yun et al., 2008
2	TLR-2/NF-κB p65 inhibitory pathway	Parkinson's disease	Chao et al., 2016
3	Complement inhibition	Coagulation	Thuong et al., 2006
4	PPARγ-C/EBPβ- inhibition mechanisms	Osteoporosis	Li et al., 2014
5	ERK pathway	Neurodegeneration	Soumyanath et al., 2012
6	ERK/p38MAPK pathway	Breast cancer	Hsu et al., 2005
7	NDR1/2 dependent p21WAF1/CIP1 activity	Hepatic cancer	Chen et al., 2014
8	Myeloid differentiation factor-88 inhibitor	Alcoholic hepatitis	Wei et al., 2013
9	TGF-β1 inhibition	Keloid	Bian et al., 2013
10	p38/ERK1/2/NF-κB inhibitory activity	Cardiac hypertrophy	Si et al., 2014
11	Ras/Raf/MEK/ERK inhibitory pathway	Lung cancer	Wang et al., 2013
12	TGF-β1/Smad signaling inhibition	Wound healing	Wu et al., 2012
13	Nerve growth factor induction	Neurodegeneration	Soumyanath et al., 2005
14	IKKβ kinase inhibitory activity	Anti-inflammatory	Patil et al., 2015
15	TGF-β/Smad3 pathway	Renal fibrosis	Meng et al., 2015
16	Smad7 agonist/Smad3 antagonist	Renal fibrosis	Meng et al., 2015
17	TGF-β1/Smad signaling inhibition	Liver fibrosis	Tang et al., 2012
18	NF-κB/STAT3/ERK inhibition	Neurodegeneration	Park et al., 2017
19	PPARγ/NLRP3 inflammasome inhibition	Hepatic injury	Xu et al., 2017
20	AMPK/GSK3β activation pathway	Hepatic failure	Lv et al., 2017
21	MAPKs inhibition pathway	Brain injury	Wen et al., 2017
22	PI3K/Akt/mTOR activation pathway	Parkinson's disease	Nataraj et al., 2017
23	MAPK/P38 and JNK/ERK inhibition	Parkinson's disease	Nataraj et al., 2017
24	Nrf2 signaling activating Akt/ERK pathway	Liver injury	Qi et al., 2017
25	AMPKα activation pathway	Cardiac hypertrophy	Ma et al., 2016
26	mTOR/ERK inhibitory pathway	Cardiac hypertrophy	Ma et al., 2016
27	TGF-β1/Smad/ERK1/2 inactivation	Lung fibrosis	Dong et al., 2017
28	NLRP3 inflammasome inhibition	Lung fibrosis	Dong et al., 2017
29	Nrf2/NLRP3 inflammasome inhibition	Lung injury	Jiang et al., 2016b
30	Nrf2/HO-1/NLRP3 inflammasome pathway	Spinal cord injury	Jiang et al., 2016a
31	NLRP3 inflammasome inhibition	Colitis	Guo et al., 2015
32	ERK/p38 inhibitory pathway	Leukemia	Wu et al., 2015

## Therapeutic potential of asiatic acid

**Figure 2:**
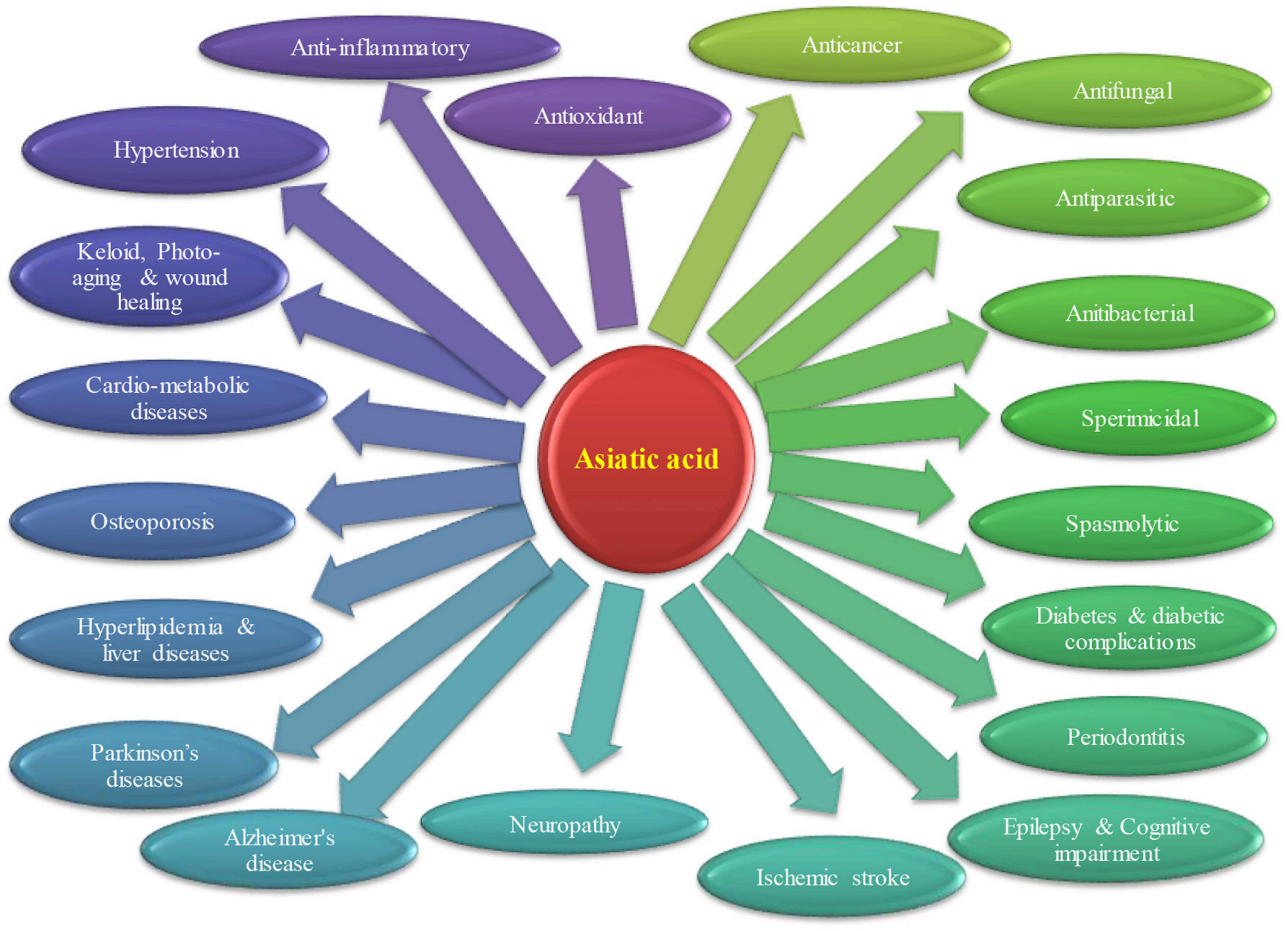
Therapeutic potential of asiatic acid in different diseases.

Convincing number of studies have demonstrated the pharmacological activities and therapeutic potential of AA that is depicted in Figure 2. Briefly, the therapeutic potential in each disease are discussed below.

## Asiatic acid in renal diseases

Renal fibrosis, which presents mainly as a tubule interstitial fibrosis in the kidney is a common pathogenic response to the injuries in chronic kidney disease that mainly involves tubular epithelial cells, myofibroblasts, endothelia, and inflammatory cells (Xu et al., 2013). TGF-β1 is a pleiotropic and multifunctional cytokine and activation of TGF-β1-mediated signaling serves as a master switch and plays a crucial role in fibrosis. Imbalance in TGF-β/Smad signaling, reflected by activated Smad3 and inhibited Smad7 is a central mechanism of tissue fibrosis. AA attenuated unilateral ureteral obstruction-induced tubule interstitial renal fibrosis in mice (Xu et al., 2013). AA ameliorated increase tubular fibrosis by diminishing fibroblast activation and accumulation of extracellular matrix mediating Smad-dependent TGF-β1 signaling pathway.

Further, AA showed to inhibit renal fibrosis in a mouse model of obstructive nephropathy modulating the TGF-β/Smad3/Smad7 signaling mechanisms (Meng et al., 2015). AA in combination with naringenin, a polyphenolic molecule of citrus fruits, inhibited Smad3 phosphorylation and transcription. AA behaved as a Smad7 agonist and naringenin as a Smad3 agonist, produced an additive effect and showed this combination as a novel agent for the treatment of chronic kidney disease (Meng et al., 2015). In a recent study, AA attenuated doxorubicin-induced renal injury by mitigating oxidative stress, inflammation by Nrf2 pathway (Kamble et al., 2017).

## Asiatic acid in inflammatory bowel disease

AA attenuated dextran sulfate sodium, a chemical colitogen induced ulcerative colitis in mice (Guo et al., 2015). The potential of AA in inflammatory bowel diseases was confirmed by improved disease activity index, inhibition of pro-inflammatory cytokines and reduced caspase-1 activation in peritoneal macrophages. AA also prevented the secretion of IL-1β, activation of caspase-1 inflammasome. Additionally, AA was found to inhibit free radical generation and maintained mitochondrial membrane potential (Guo et al., 2015).

## Asiatic acid in epilepsy, depression, and associated complications

Epilepsy is amongst the most common neurological conditions involving oxidative stress, glutamate toxicity, and neuroinflammation mainly in the hippocampus. The anticonvulsant agents used for the treatment often show adverse effects, intolerance, and a lack of efficacy. AA was found to reduce severity and frequency of seizures, kainic acid-induced convulsions in mice by mitigating oxidative stress, neuroinflammation, and apoptotic cell death (Wang et al., 2017). AA has been shown to reduce the production of inflammatory cytokines and mediators such as cyclooxygenase-2 and NF-κBp50/65 in the hippocampus. AA reduced free radical generation, restored glutathione content, enhanced activity of glutamine synthetase, reduced levels of glutamate, and increased level of glutamine in the hippocampus. AA also affected cell death machinery by improving the expression of Bcl-2 and reducing the expression of Bax. In NGF-differentiated PC12 cells, AA improved the viability of cells and preserved integrity of the plasma membrane. The anticonvulsant drugs used in epilepsy are known to affect memory and learning over time. In recent years, AA has been patented for treating dementia and can be used for cognitive disorders, cerebrovascular and central nervous system diseases as a cognition enhancer by the Hoechst Aktiengesellschaft (EP0383171 A2). Dementia diagnosed clinically based on progressive decline in cognition that appears frequently or is a common accompaniment in the neurodegenerative diseases (Cunningham et al., 2015).

AA was found to ameliorate memory and cellular side effects of valproic acid in rats and affected the Ki-67 and BrdU proteins, markers of cell proliferation (Umka Welbat et al., 2016). AA treatment reversed valproic acid-induced impaired spatial working memory, proliferation of the cells and salvaged subgranular zone of the hippocampal dentate gyrus as valproic acid adversely affects neural stem cell proliferation and differentiation (Welbat et al., 2016). Lee et al. (2000), synthesized about 36 derivatives of AA and screened them for neuroprotective efficacy in rat cortical neurons challenged with glutamate (Lee et al., 2000). Some derivatives were found to mitigate glutamate neurotoxicity by inhibiting glutathione depletion and NO overproduction along with maintenance of antioxidant mechanisms. In another study, AA showed neuroprotection in human neuroblastoma cells (SH-SY5Y) and in learning and memory in mice (Xu et al., 2012). AA was found to ameliorate glutamate neurotoxicity in a concentration-dependent manner as evidenced by decreased apoptosis and ROS production, stabilized mitochondrial membrane potential and enhanced PGC-1α and Sirt1 expression. AA also prevented neuronal damage of the pyramidal layer in the CA1 and CA3 regions of hippocampus, restored antioxidants and attenuated cognitive deficits in mice (Xu et al., 2012). Further, in two-electrode voltage-clamp technique AA selectively produced negative modulation of different GABA_A_ receptor subtypes expressed in *Xenopus laevis* oocytes (Hamid et al., 2016). The activity on α5-containing GABA_A_ receptors showed the role of this receptor in cognition and memory that provides a basis of its traditional use in the treatment of cognitive disorders and anxiety. These studies suggest that AA and its derivatives are promising for memory and cognition enhancement. Thus, AA can be used either alone or as an adjuvant in neuropsychiatric diseases including epilepsy as well as learning and memory impairment.

## Asiatic acid in diabetes

AA isolated from ethyl acetate fraction of extract of the *Lagerstroemia* leaves exhibited weak alpha-amylase and alpha-glycosidase inhibitory activity in assays (Hou et al., 2009). AA exerts its anti-hyperglycemic effects by preserving and restoring the number of beta cells and their function in rodent models of diabetes (Liu et al., 2010). Simultaneously, AA also enhanced serum insulin along with salvage of pancreatic beta cells in diabetic rats. Mechanistically, it promoted cell survival machinery by promoting activation of Akt kinase and Bcl-xL in the pancreatic islets. The antidiabetic activity of AA was shown mediating PI3K/AKT/GSK-3β signaling mechanism in high-fat diet fed db/db mice (Sun et al., 2017). AA attenuated rise in expression of PI3K, AKT, insulin receptors, and insulin receptor substrate-1 and downregulated GSK-3β and glucose-6-phosphatase. The observed antidiabetic effects evidenced by normalized glucose and lipid levels and glycogen synthesis along with histological salvage. Ramachandran and Saravanan (2013) has demonstrated that oral administration of AA to diabetic rats decreased the levels of blood glucose, increased insulin levels and corrected glycosylated hemoglobin as well as hemoglobin. AA also decreased the activities of glucose-6-phosphatase and fructose-1,6-bisphosphatase and increased the activities of hexokinase, pyruvate kinase, glucose-6-phosphate dehydrogenase involved in carbohydrate metabolism. Additionally, it also increased the levels of liver glycogen along with normalizing the activities of liver injury markers enzymes in diabetic rats. The anti-hyperglycemic effect of AA was equivalent to glibenclamide, a standard anti-hyperglycemic drug clinically used in the treatment of diabetes.

Furthermore, AA normalized the levels of blood glucose, improved insulin levels, restored antioxidant defense system, and reduced lipid peroxidation. Further, at the molecular level, AA augmented insulin receptors, IRS-1/2, PI3K, Akt, and glucose transporter 4 (GLUT4) proteins involved in glucose homeostasis (Ramachandran and Saravanan, 2015). Further, in molecular docking AA showed affinity against HMG-CoA reductase, an enzyme involved in cholesterol biosynthesis with a binding energy of 11.8122 kcal/mol. In addition, AA also resored the levels of blood glucose, insulin, lipid profile, atherogenic index, and improved the levels of high-density lipoprotein in diabetic rats. The observed beneficial effects of AA in this study were compared with glibenclamide (Ramachandran et al., 2014). Using the Goto-Kakizaki (GK) rats, a model of spontaneous type 2 diabetes characterized by a progressive loss of beta islet cells with fibrosis. Wang et al. (2015) has demonstrated that AA prevented islets dysfunction by lowering blood glucose levels and improving fibrosis of islets in GK rats.

Integrating different studies together, AA appears to be a promising molecule for type 1 as well as type-2 diabetes and hyperlipidaemia, which occur commonly in diabetic subjects. The underlying mechanism could be an improved glucose homeostasis by enhanced antioxidant defense as well as modulation of proteins, GLUT4 and Akt involved in glucose metabolism in skeletal muscles. AA at 0.1 or 0.2% concentration fed to diabetic mice protected the diabetic heart by reducing glycative injury and coagulation components (Hung et al., 2015). AA reduced the levels/activities of plasma glucose, creatine phosphokinase, lactate dehydrogenase, and restored HbA1c levels in diabetic mice. It improved the levels of glutathione, reduced ROS production, N(ε)-(carboxymethyl)-lysine, pentosidine, methylglyoxal, pro-inflammatory cytokines, and chemokines in diabetic mice. AA also reduced von Willebrand factor, fibrinogen levels, factor-VII and maintained circulating antithrombin-III and protein-c activities in the plasma. AA also decreased the activities of NADPH oxidase and aldose reductase and expressions of glyoxalase 1, NF-κB-p65, NF-κB-p50, and the receptors of advanced glycation products along with the expressions of p-p38 and p-ERK1/2 in diabetic heart. In a study, wherein numerous pentacyclic triterpenoids were evaluated for their activites on rabbit muscle glycogen phosphorylase a (GPa). In SAR studies, the presence of a sugar moiety in triterpene saponins is attributed to its reduced activity. These saponins appear as a potential prodrugs of natural origin and they possess higher water-soluble property than the corresponding aglycones (Wen et al., 2008).

## Asiatic acid in cardiac hypertrophy

Cardiac hypertrophy occurs as a compensatory mechanism against pressure overload in hypertension or aortic stenosis characterized by an increase in the ventricular mass (Frohlich and Susic, 2012). It is an independent risk factor for heart failure or sudden cardiac death therefore that effective therapeutic agents for prevention and treatment of cardiac hypertrophy are needed (Frohlich and Susic, 2012). The effects of AA on cardiac hypertrophy and underlying mechanism were studied using pressure overload-induced mouse model of cardiac hypertrophy and cultured neonatal cardiomyocytes stimulated with TGF-β1 which triggers pathological cardiac hypertrophy (Si et al., 2014). AA has been shown to attenuate cardiomyocyte hypertrophy by reducing the surface area and by inhibiting the expressions of atrial natriuretic peptide as well as p38, p-ERK1/2 phosphorylation, and NF-κB binding activity in cardiomyocytes (Si et al., 2014). AA reduced the activation TGF-β1 signaling in the pressure-overload mice model of cardiac hypertrophy. In another study, Xu et al. (2015) has revealed the efficacy of AA against transverse aortic constriction-induced cardiac hypertrophy in C57BL/6 mice and cultured neonatal cardiomyocytes AA inhibited IL-1β-related hypertrophic signaling that suppressed cardiac hypertrophy. Similar observations were noted in another study in a transverse aortic constriction mice model mimicking the progression of hypertrophy and heart failure (Si et al., 2015). AA attenuated cardiomyocyte apoptosis by blocking the activation of both death receptor and mitochondrial dependent apoptotic signaling and ameliorated interstitial fibrosis and inflammation by blocking the activation of both transforming growth factor-β1/Smad and IL-6 (Si et al., 2015). Further, it appears to be protective against coronary artery ligation-induced MI in rats. AA treatment has been shown to improve cardiac function as assessed by echocardiography, reduced inflammatory cytokines and interstitial fibrosis (evidenced by reduced collagen II and III expressions). AA also prevented the left ventricular remodeling in the infarct area of the ischemic myocardium by inhibiting phosphorylation of p38, MAPK, and ERK1/2 (Huo et al., 2016).

Kalyanavenkataraman et al. (2016) showed another cardioprotective mechanism wherein AA appears to be the most potent agent in inhibiting carbonic anhydrase II with an IC_50_ of 9 μM as well as cytosolic activity in H9c2 cardiomyocytes accompanied by decreased intracellular levels of Ca^+2^, acidification and mitochondrial membrane depolarization. Increased activities of carbonic anhydrase II is associated with cardiac hypertrophy and heart failure. In another study, AA attenuated doxorubicin-induced oxidative stress and inflammation in the heart, liver, and kidney by upregulating Nrf2 protein expression (Kamble and Patil, 2018). Recently, AA showed to inhibit pressure overload or angiotensin II induced hypertrophic responses by suppressing collagen accumulation mediated cardiac fibrosis (Ma et al., 2016). Mechanistically, the protective effects of AA were mediated by the activation of AMPKα that participates in the pathogenesis of cardiac hypertrophy and inhibition of the mammalian target of rapamycin (mTOR) pathway and ERK.

## Asiatic acid in cancer

Cancer is a disease results from both genetic and epigenetic changes and often treated by either chemotherapy or radiation therapy or a combination of both. Conventional agents used in cancer chemotherapy are often toxic not only to tumor cells but also to the normal cells and limit their clinical use. Due to favorable safety and efficacy, novel natural compounds remain an alternative to synthetic compounds. In past few years, phytochemicals received more interest in research related to cancer drug discovery and development along with ethnopharmacological and reverse pharmacological approaches due to their potential recognition as source of numerous drugs, wide availability, accessibility, and perceived time tested acceptance and safety over synthetic compounds. Many phytochemicals have shown to exert anticancer, chemopreventive, or chemosensitizer effect or act as adjuvants in attenuating adverse effects caused by chemotherapeutic drugs in cancer treatment (Mann, 2002). Despite of their chemotherapeutic potential, the poor solubility, stability, bioavailability, and target specificity affecting their pharmaceutical development and make their clinical application unrealistic. Many of the anticancer drugs are of natural origin such as vinca alkaloids, taxanes, camptothecins that elicit cytotoxic activity contributing to effective cancer treatment (Jiang and Liu, 2008). In past few years, numerous pre-clinical and clinical studies demonstrated the anticancer potential of AA itself and validated the traditional claims of anticancer potential of many plants containing AA as a major ingredient used in traditional medicines. The anticancer and chemopreventive efficacy of several medicinal plants is attributed mainly to the presence of AA. The anticancer activity and underlying pharmacological and molecular mechanisms of AA in different preclinical studies are summarized in Table 7.

**Table 7:** The anticancer activities and mechanism of Asiatic acid in different cancer types.

**Asiatic acid & derivatives**	**Cell lines/cancer models**	**Mechanisms/effects observed**	**Cancer type**	**References**
AA	HepG2	↓ intracellular Ca^2+^levels ↑ apoptosis and p53 expression	Liver cancer	Lee et al., 2002
AA	SK-MEL-2	↓ viability and ↑ apoptosis ↑ ROS levels and Bax but not Bcl-2 ↑ activation of caspase-3 activity	Skin cancer	Park et al., 2005
AA	HT-29	↑ cytotoxicity, DNA fragmentation, apoptosis, and caspase-3 activation augmenting effect of irinotecan ↓ Bcl-2 and Bcl-xL proteins ↑ apoptosis via caspase-3 activation	Colon cancer	Bunpo et al., 2005
AA	PPC-1	↑ caspase-dependent and independent cell death ↑ activation of caspase 2,3, & 8 but not 9 ↑ Ca^2+^ release and dilatation of endoplasmic reticulum	Prostate cancer	Gurfinkel et al., 2006
AA	U-87 MG	↑ dose- and time-dependent apoptosis and necrosis ↓ mitochondrial membrane potential ↑ Ca^2+^ and caspase-9 and−3	Glioblastoma	Cho et al., 2006
AA	TPA-DMBA-initiated ICR mice	↓ NO generation and expression of iNOS and COX-2	Skin tumor	Park et al., 2007
AA	SW480	↓ cancer cell proliferation ↑ mitochondrial membrane permeability and cytochrome c release from mitochondria into cytosol ↑ caspase-3, 9 activity and poly(ADP-ribose) polymerase results in apoptosis	Colon cancer	Tang et al., 2009
AA	Human umbilical vein endothelial cells (HUVEC) and human brain microvascular endothelial cells (HBMEC), human glioma cells (LN18 & U87-MG)	↓ growth and capillary tube formation ↑ apoptosis by activating caspases 3 & 9 modulates expression of apoptosis regulators Bad, survivin and pAkt-ser473 ↓ cellular and secreted VEGF levels dose-dependent antiangiogenic effect	Gliomas	Kavitha et al., 2011
AA derivatives (120)	HeLa, HepG2, BGC-823, and SKOV3	↓ cell growth potent than AA substitution of amide group at C-28 shows potent cytotoxicity than AA comparable to gefitinib and etoposide	Many cancer types	Meng et al., 2012
AA derivatives (5)	A549 and PC9/G	↓ cell growth potently than AA ↓ proliferation via down-regulation of the Ras/Raf/MEK/ERK pathway and cell cycle arrest at G1/S and G2/M	Lung cancer	Wang et al., 2013
AA	HepG2	↑ p21WAF1/CIP1 expression but no p21WAF1/CIP1 mRNA ↑p21WAF1/CIP1 with ↓ phosphorylation (ser-146) of p21WAF1/CIP1 ↓ NDR1/2 kinase and proliferation ↑ stability of p21WAF1/CIP1	Liver cancer	Chen et al., 2014
AA	Colon cells	↓ IL-8 production	Colon cancer	Yan et al., 2014b
AA		↓ tyrosinase mRNA expression by inhibiting microphthalmia-associated transcription factor (MITF)	Melanoma	Kwon et al., 2014
AA	A549	↑ miR-1290, which sensitizes cells to AA cytotoxicity & ↓ viability & cell cycle progression negatively regulates BCL2 expression	Lung cancer	Kim et al., 2014
AA	MGC-803, NCI-H460, HepG2, Hela and 7404	anticancer drug 5-fluorouracil stronger anti-proliferative activity than AA ↑ ROS and altered anti- & pro-apoptotic proteins leading to mitochondrial dysfunction and activations of caspase-3,−9 arrested HepG2 cells in G1 stage	Many cancer types	Li et al., 2014
AA	Human GBM cell (LN18, U87MG, and U118MG) ectopic U87MG xenograft implantation in mice	↓ cell viability, better efficacy than temozolomide at equimolar doses decreased tumor volume in mice without toxicity decreased orthotopic U87MG xenografts growth in nude mice in magnetic resonance imaging crosses blood-brain barrier induced apoptotic death by modulating the protein expression of several apoptosis regulators (caspases, Bcl2 family members, and survivin) induced ER stress (increased GRP78 and calpain, and decreased calnexin and IRE1α expression) enhanced free intra-cellular calcium, and damaged cellular organization in GBM cells	Glioblastoma multiforme	Kavitha et al., 2015
AA	HL-60 leukemia cell line cells	blocked cell growth in a dose- and time-dependent manner induced apoptosis in a dose-dependent manner downregulated anti-apoptotic proteins Bcl-2, Mcl-1 and survivin were by AA in a dose-dependent manner inhibited ERK and p38 phosphorylation in a dose-dependent manner, while JNK phosphorylation was not affected	Blood cancer	Wu et al., 2015
AA modified compounds at the C-2, C-3, C-23, and C-28	HeLa, HepG2, B16F10, SGC7901, A549, MCF7, and PC3), larval zebrafish model	potent cytotoxic & anti-angiogenic than AA markedly better anti-tumor activities than both AA and other derivatives, with similar stability as its parent compound AA	Many cancer types	Jing et al., 2015
AA	Virtual screening, cell free medium	NF-κB inhibitory down regulators of IKKβ phosphorylation	Inflammation in cancer	Patil et al., 2015
AA loaded Solid lipid nanoparticles (SLNs)	U87 MG and SVG P12	displayed more favorable drug release profile and higher cytotoxicity cellular uptake of SLNs appear preferentially facilitated by energy-dependent endocytosis concentration-dependent apoptosis	Brain cancer	Garanti et al., 2016
Fluorinated Asiatic Acid (AA) derivatives	HeLa and HT-29 cell lines, MCF-7, Jurkat, PC-3, A375, MIA PaCa-2 and BJ HeLa cell line, non-tumor BJ human fibroblast cell line	concentration dependent antiproliferative activity than AA most active compounds have a pentameric A-ring containing an α,β-unsaturated carbonyl group ↑ cell cycle arrest in G0/G1 stage, ↑ p21(cip1/waf1) and p27(kip1) ↓ cyclin D3 and Cyclin E, ↑ caspases-8 and 3 & cleavage of PARP ↑ Bax and ↓ Bcl-2 and cleavage Bid into t-Bid extrinsic and intrinsic apoptotic pathways	Many cancer types	Gonçalves et al., 2016
AA	HepG2	anti-proliferative activity against HepG2	Liver cancer	Shi et al., 2016
AA	SKOV3 and OVCAR-3	↓ 50% in the viability of cells, ↓ colony formation of cells by 25–30% cell cycle arrest at the G0/G1 phase ↑ 7- to 10-fold increase in apoptosis ↓ PI3K, Akt, and mTOR phosphorylation	Ovarian cancer	Ren et al., 2016
N-(2α,3β,23-acetoxyurs-12-en-28-oyl)-l-proline methyl ester (AA-PMe)	SGC7901 and HGC27 human gastric cancer cells human gastric mucosa epithelial cells (GES-1)	↓ cell proliferation dose-dependently ↑ cell cycle arrest in G0/G1 phase, blocked G1-S transition ↓ cyclin D1, CKD4, and phosphorylated retinoblastoma protein ↑ cyclin-dependent kinase inhibitor P15 ↑ apoptosis affecting Bcl-2, Bax, c-Myc, and caspase-3 ↓ migration and invasion reducing MMP-2 and 9	Gastric cancer	Jing et al., 2016
AA	Ovarian cancer cells (A2780)	↑ cytotoxicity by inducing apoptosis	Ovarian cancer	Sommerwerk et al., 2016
AA	1,2-dimethylhydrazine (DMH)-induced colon carcinogenesis in male Wistar rats	↓ incidence of polyps & aberrant crypt foci ↑ antioxidants & ↓ lipid peroxidation in liver alterated activity of biotransforming enzymes & histological improvement	Colon cancer	Siddique et al., 2017
AA proline methyl ester derivative (AA-PMe)	Gastric cancer cells	↓ STAT3 activation mediated by blockade of Janus-activated kinase 2 dose dependently regulated expression of STAT3-modulated gene products including cyclin D1, Bax, Bcl-2, c-Myc and MMP-2 and 9	Gastric cancer	Wang et al., 2017
AA derivatives	Cancer cell lines (HepG2 and SGC7901)	compounds I_2_, I_6_, and II_6_ potent cytotoxic activity than that of the positive control paclitaxel in MTT assay docking shows interactions between compounds I_2_,I_6_,II_6_ and survivin	Liver cancer	Meng et al., 2018
AA	SVG p12 fetal glia and U87-MG grade IV glioblastoma cells under normoxic (21% O_2_) and hypoxia (1% O_2_)	cell viability, proliferation, apoptosis and wound healing cytotoxic effects on glioma cell lines and has the potential to become an alternative treatment for glioblastoma	Glioblastoma	Thakor et al., 2017
AA	Lung cancer both xenograft model in mice *in vivo* and *in vitro*	induced apoptosis and autophagy (elevated microtubule-associated protein 1 light chain 3 (LC3) and decreased p62 expression prevented mitochondrial injury, decreased expression of proliferating cell nuclear antigen	Lung cancer	Wu et al., 2017

**Figure 3:**
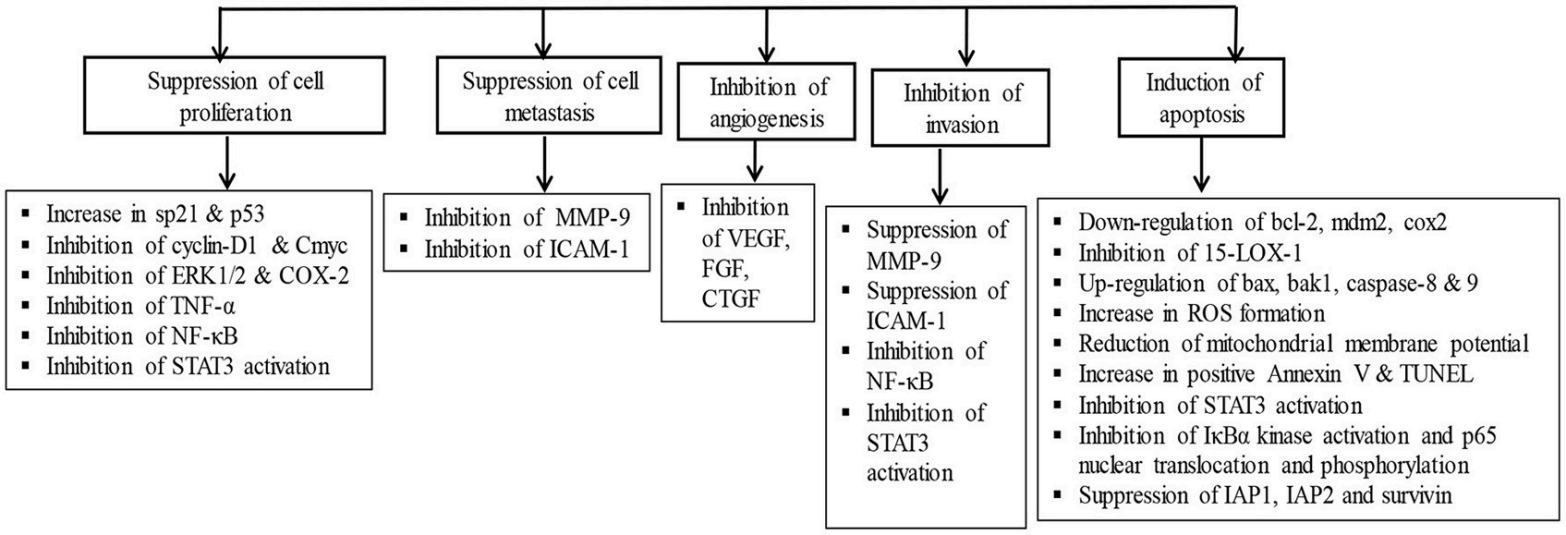
The chemopreventive and anticancer mechanism of asiatic acid in different cancer cell lines and animal studies.

Recently, several studies have demonstrated the pro-apoptotic and cell growth inhibitory activity of AA in the liver, breast, skin, brain, and gastrointestinal tumor cells (Lee et al., 2002; Hsu et al., 2005; Park et al., 2005; Cho et al., 2006; Tang et al., 2009; Kavitha et al., 2015; Wu et al., 2015). AA treatment was found to attenuate inflammation, tumor cell proliferation, and induce mitochondrial pathway of apoptosis. The anticancer effects of AA are attributed to its ability to inhibit transcription factor NF-κB, p38-MAPK, and ERK kinases in a variety of tumor cells. AA also modulates diverse signaling pathways including CDKs and cyclins which regulate cell proliferation, c-myc, EGFR, and vascular endothelial growth factor which regulate growth factors, p53 and p21 which regulate tumor suppression. Additionally, AA also affected the apoptotic mediators; Bcl-2, Bcl-xL, XIAP, caspases and death receptors, inflammatory mediators (NF-κB and COX-2), protein kinases (JNK, Akt, and AMPK), and oncogenes (MDM2). Since knowing the critical role of apoptosis in cell survival and death, the manipulation of the apoptotic process by novel agents could be the suitable candidates for the chemotherapy of cancer (Schulte-Hermann et al., 1997). AA showed to inhibit tumor progression by inhibiting cell proliferation, inducing cell cycle arrest and apoptotic cell death in numerous cancer cells and increasing the anti-angiogenic activity and sensitivity of cancer cells to the treatment with chemotherapeutic agents (Gonçalves et al., 2016). The signaling molecules targeted by AA and its anticancer mechanism in different cancer cell lines and animal models are presented in Figure 3.

The *in vitro* antitumor activity of AA derivatives against MGC-803, NCI-H460, HepG2, Hela, and 7404 cancer cell lines were compared with clinically available anticancer drug 5-fluorouracil (5-FU). The new derivatives showed more potent anti-proliferative activity than AA. They also caused induction of ROS generation, activation of caspase-3 and -9 to induce apoptosis and regulate mitochondrial anti- and pro-apoptotic proteins and arrested cell cycle in G1 stage in HepG2 cells (Li et al., 2014). Jian-Fei et al. showed that insertion of an amide bond in C-28 combined and a carbonyl moiety at C-11 enhances anticancer action. Another study demonstrated that substitution of amide group at C-28 and acetylation of hydroxyl groups at C-2, C-3, and C-23 provides derivatives with more potent inhibitory action on cell growth in many cancer cells than AA. Furthermore, AA derivatives formed following modification of A-ring showed improved cytotoxicity against melanoma (Malme-3M cells) and neoplasm P388D1 (Gonçalves et al., 2016). AA derivatives synthesized following modification at C-2, C-3, C-23, and C-28 positions elicit potent anticancer action in different cancer cell lines (HeLa, HepG2, B16F10, SGC7901, A549, MCF7, and PC3) and inhibited angiogenesis in larval zebrafish, an *in vivo* model (Jing et al., 2015). These derivatives appear to be more potent as compared to AA with similar stability as its parent compound AA. Meng et al. (2012) synthesized and confirmed structures of 12 novel derivatives of AA and found many of them caused inhibition of cell growth superior than AA.

Jing et al. (2016) synthesized many AA derivatives to improve its therapeutic potency and found N-(2α,3β,23-acetoxyurs-12-en-28-oyl)-l-proline methyl ester (AA-PMe) more potent anticancer candidate for gastric cancer compared to AA. This new derivative showed potent anticancer activity following dose-dependently inhibition of cell proliferation in human gastric cancer cells (SGC7901 and HGC27) without affecting human gastric mucosa epithelial cells (GES-1). It decreased cyclin D1, cyclin-dependent kinase CKD4, and phosphorylated retinoblastoma protein along with increased cyclin-dependent kinase inhibitor P15, that leads to cell cycle arrest in G0/G1 phase and inhibition of G1-S transition. AA-PMe also enhanced apoptosis and reduced migration and invasion of cells by activating apoptotic proteins Bcl-2, Bax, c-Myc, and caspase-3 and inhibiting MMP-2 and -9. AA upon microbial transformation provides novel and potent derivatives which exhibit cytotoxic effects in several human cancer cell lines (Guo F. F. et al., 2012). Despite the fact that many studies have demonstrated the anticancer property of AA, more *in vivo* studies are required to establish and prove the antiangiogenic and antimetastatic potential of AA. In the past few years, many derivatives of AA have been synthesized and found better than AA itself in terms of efficacy, stability, and provide a resource for synthetic development for further development. Though, majority of them showed potent anticancer but more studies are needed in detail for their pharmacological basis in therapeutics, safety, and regulatory toxicology.

## Asiatic acid in non-small cell lung cancer

Non-small cell lung cancer accounts for more than 80% of total pulmonary malignancies (Zalcman et al., 2010). For pharmaceutical development, five AA derivatives were synthesized and assessed for their effect on growth in non-small cell lung cancer cells; A549 and PC9/G. Among them, four derivatives strongly inhibited cell growth in a concentration and time-dependent manner as compared to AA. Compound A3 has been shown to promote antiproliferative and cell cycle dysregulation effects. Affymetrix Gene Chip^®;^ Human Genome U133 array was used to monitor transcriptome differences in order to regulate cellular gene manifestation. Alteration of *1121* genes in A549 and *1873* genes in PC9/G has shown following treatment with AA. AA effects on proliferation were mediated by the downregulation of Ras/Raf/MEK/ERK pathway and cell cycle arrest at G1/S and G2/M (Wang et al., 2013). Ras/Raf/MEK/ERK and Ras/PI3K/PTEN/Akt/mTOR signaling pathways are known to play key roles in facilitating the proliferative signal transmission from membrane-bound receptors and relay extracellular information through an interaction with various cellular proteins within the nucleus to control gene expression (Steelman et al., 2011).

AA elicits cytotoxicity mediated by microRNA (miR)-1290 that regulates apoptotic cell death, cell viability and cell cycle progression in A549 cells (Kim et al., 2014). AA also induced apoptosis, loss of mitochondrial membrane potential and generated free radicals along with improved microtubule-associated protein 1 light chain 3 (LC3) and reduced expression of p62 (Wu et al., 2015). Orally administered AA reduced the tumor volume and expression of proliferating cell nuclear antigen by promoting apoptosis in the mouse lung cancer xenograft model.

## Asiatic acid in melanoma

Melanoma is the deadliest form of skin cancers and about 10% cases occur in a familial context involving cyclin-dependent kinase inhibitor 2A as a main high-risk gene for melanoma (Potrony et al., 2015). Park et al. (2005) has demonstrated the time- and dose-dependent anticancer activity of AA in skin cancer by inducing apoptosis and decreasing cell viability in human melanoma cells (SK-MEL-2). Though, AA was found to increase the levels of ROS, Bax, and caspase-3 expressions in a concentration-dependent manner, it failed to raise p53 levels present in the cells. In another study, the antiproliferative activity of AA has been demonstrated in murine melanoma cells; B16F10 (Yoshida et al., 2005). Taken together, AA appears as a promising agent for human skin cancer.

## Asiatic acid in breast cancer

Breast cancer is a common cancer in women with an upward trending incidence especially with increasing age. The pathology and biology of breast cancer seems to be different in the elderly, often resulting in under treatment and thus in higher rates of recurrence and mortality (Dimitrakopoulos et al., 2015). AA inhibited cell growth by activation of p38 and ERK1/2 kinases, reduced survival of cancer cells, caused cell cycle arrest in S-G2/M phase and induced apoptosis in breast cancer cells; MCF-7 and MDA-MB-231 (Hsu et al., 2005). AA also accelerated the interaction between p21 and Cdc2 and reduced expression of Cdc2, Cdc25C, cyclinB1, and cyclinA that inhibited cell cycle progression. It also demonstrated the role of p38 pathway in cell cycle arrest and ERK1/2 cascade in apoptosis, but not in cell cycle regulation. AA found promising for chemopreventive purposes in breast cancer, though *in vivo* studies are yet to translate the *in vitro* findings.

## Asiatic acid in prostate cancer

Prostate cancer relies on androgen-dependent signaling for initiation, progression and development. In PPC-1 prostate cancer cells, AA was found to induce caspase-dependent and independent cell death by activation of caspases 2, 3, 8, and 9. AA treatment disrupted endoplasmic reticulum and altered calcium homeostasis (Gurfinkel et al., 2006). However, this preliminary study is yet to be confirmed *in vivo*.

## Asiatic acid in multiple myeloma

Multiple myeloma, a hematological cancer involves malignant proliferation of monoclonal plasma cells that leads to hypercalcemia, renal dysfunction, anemia, and bone disease thereby promoting organ failure (Prideaux et al., 2014). AA showed inhibition of cell proliferation and growth by arresting the progression of cell cycle in a time- and concentration-dependent manner and led to G_2_/M phase and reduced expression of focal adhesion kinase (FAK) and phosphorylated-FAK mediated signal transduction in multiple myeloma cells; RPMI 8226 (Zhang H. R. et al., 2013; Zhang J. et al., 2013). Furthermore, reduced FAK expression also indicated the antitumor mechanism of AA.

## Asiatic acid in hepatoma

Hepatocellular carcinoma is the one of the major cancer in occurrence with high morbidity and mortality. Among several cellular and molecular events that contribute to tumor initiation, progression and metastasis, cell death by apoptosis plays a major factor in this pathogenesis (Mizuguchi et al., 2016). In growth, differentiation, or senescence, accumulation of *p53* related oncogenic signals play a key role in the preserving tissue homeostasis (Schulte-Hermann et al., 1997). AA was found to regulate various signaling cascades that occur in hepatoma HepG2 cells. AA triggers apoptosis *via* increasing intracellular Ca^2+^ levels that leads to an enhanced p53 expression (Lee et al., 2002). AA also exhibits a potent antioxidant effect in HepG2 cells challenged with tert-butyl hydroperoxide (Qi). Additionally, AA causes activation of *Nrf2* along with antioxidant genes such *HO-1, NQO-1*, and *GCLC* as well as *ARE* and reduced *Keap1*. The observations were reconfirmed in Nrf2 knockouts. Further, a concomitant activation of Akt and ERK signals was observed with Nrf2 activation in HepG2 cells.

In another study, AA showed to inhibit HepG2 cells proliferation by suppressing NDR1/2 kinase expression and promoted stability of p21WAF1/CIP1 that led to amelioration of NDR1/2 dependent phosphorylation of p21WAF1/CIP1 (Chen et al., 2014). NDR kinase family proteins are implicated in controlling G1/S transition downstream in mitotic process of cell growth (Cornils et al., 2011), whereas, p21Waf1/CIP1 proteins participate in cell cycle control, blocking the transition from phase G1 to S (Pérez-Sayáns et al., 2013).

## Asiatic acid in glioma

Glioma is the one of the serious tumor of the central nervous system that originates from astrocytes, oligodendrocytes, and neural stem cells. Malignancy of glioma is often related to high mortality and is unaffected by conventional management and is associated with a poor prognosis (Onishi et al., 2011).

AA was found to improve the outcome in patients with glioma (Kavitha et al., 2011). The glioma cells exhibit an enhanced formation of capillary tubes in both human umbilical vein endothelial cells (HUVEC) and human brain microvascular endothelial cells (HBMEC). AA potently suppressed VEGF secretion and its cellular level in glioma cells in Matrigel plug assay in a dose-dependent manner. AA is also known for its beneficial effects in neurological disorders with negligible side effects and good bioavailability along with BBB permeation (Mato et al., 2011; Shinomol, 2011). AA may be important in glioma by antiangiogenic mechanism.

## Asiatic acid in glioblastoma

Glioblastoma also known as glioblastoma multiforme is an aggressive brain tumor that remains incurable, and thus requires novel therapeutic agents (Furnari et al., 2007). AA reported to induce a dose- and time-dependent cell death *via* both apoptosis and necrosis in human glioblastoma cells (U-87 MG). AA caused cell death associated with suppressed mitochondrial membrane potential, activated caspase-9 and -3 and raised the levels of intracellular Ca^2+^ mediating apoptosis and necrosis, with a predominance of necrotic cell death (Cho et al., 2006).

In another study, AA suppressed viability of human glioblastoma multiforme cells (LN18, U87MG, and U118MG) and observed superior than temozolomide (Kavitha et al., 2015). In a recent study, oral administration of AA reduced tumor volume following ectopic xenograft implantation (U87MG) in mice without toxicity. It further decreased xenograft's growth in nude mice and appeared bioavailable in the plasma and brain. AA induces apoptotic cell death by modulating various regulators of apoptosis such as caspases, Bcl2 family members and survivin. The therapeutic targeting of survivin was based on its role in tumor growth and drug resistance by promoting survival of cancer cells. AA derivatives promoted apoptosis by downregulation of survivin protein (Meng et al., 2018).

Furthermore, AA also induced ER stress and damaged cellular organization in GBM cells as evidenced by raised free intracellular calcium, GRP78, and calpain along with reduced calnexin and IRE1α expression (Kavitha et al., 2015). Rafat et al. (2008) showed that micellar aggregation property of AA leads to cytotoxicity against human small cell carcinoma and glioblastoma cells within the normal range of IC_50_. The effect of AA appears to be cell type-specific, as in colon cancer, AA exerts apoptotic mode of cell death on RKO cells. AA attenuated cell proliferation, viability, cell death, and wound healing in fetal glia (SVG p12) and grade IV glioblastoma cells; U87-MG under hypoxic conditions and found comparable to standard drug; cisplatin (Thakor et al., 2017). These reports have revealed that AA holds a promising future in GBM.

## Asiatic acid in colon cancer

AA elicited chemopreventive activity against colon cancer in rats induced by 1,2-dimethylhydrazine (DMH). AA reduced polyps and aberrant crypt foci and salvaged colonic tissues following reduction in lipid peroxidation and restoration of antioxidant defense in the colon. In another study, the protective mechanism in colon cancer was demonstrated by virtue of anti-inflammatory, antiproliferative, and pro-apoptotic properties of AA (Siddique et al., 2017). AA improved disease activity indices, decreased phase I metabolic enzymes and improved phase II metabolic enzymes and mucin in colon cancer in rats induced by DMH. AA also favorably altered apoptotic machinery and attenuated argyrophilic nucleolar organizer regions, proliferating cell nuclear antigen, cyclin D1, and activation of mast cells. AA appears to be a promising dietary agent in preventing colon cancer.

## Asiatic acid in metabolic syndrome

Metabolic syndrome (MS) is a chronic condition associated with high risk of cardiovascular diseases and is characterized by the signs of obesity, insulin resistance, impaired glucose tolerance, hypertension and dyslipidaemia (Panchal et al., 2011). MS is also considered as a primary stage of cardiovascular or cardiometabolic disease that leads to serious health concerns and increased mortality worldwide (Isomaa et al., 2001; Zimmet et al., 2001). The occurrence of excessive production of free radicals and low grade chronic inflammatory state in MS plays an important role in the development of complications including endothelial dysfunction and subsequent atherosclerosis (Li et al., 2007). Pakdeechote et al. (2014) first showed the effect of AA in attenuating MS in rats induced by feeding high-carbohydrate, high-fat diet with 15% fructose for 12 weeks. AA was found to ameliorate metabolic and hemodynamic impairment and normalized eNOS as well as iNOS expression and NOx levels in MS rats. Improvement in insulin sensitivity, hemodynamics, lipid profile, oxidative/nitrosative stress, pro-inflammatory cytokines, and recovery of eNOS/iNOS balance is found in line with the antioxidant and anti-inflammatory properties of AA. In another study, the authors reported the effect of AA on vascular structure and function, renin-angiotensin system (RAS) in high-fat high carbohydrate diet-induced MS in rats. AA attenuated metabolic derangements, hemodynamic alterations and over activation of RAS and sympathetic system. It also raised NE levels in the plasma and corrected endothelium impairment and diminished eNOS expression but did not ameliorate vascular remodeling (Maneesai et al., 2016).

Many triterpenes including AA also reported to elicit antiobesity effects. In a recent study, AA showed an anti-obesity action in high-fat food fed rat model of obesity as evidenced by the improved antioxidant activities, regulation of lipid metabolism, and insulin and leptin sensitivity in addition to reduction in body weight gain (Rameshreddy et al., 2018). AA also favorably altered expression of lipid metabolism-related genes including *acetyl CoA carboxylase, uncoupling protein-2*, and *carnitine palmitoyltransferase-1* along with improved bone mineral contents and bone mineral density. The effects were found comparable to the standard drug used in obesity management, orlistat, a pancreatic lipase inhibitor (Rameshreddy et al., 2018). Recently, AA suppressed octanoylated ghrelin levels in AGS-GHRL8 cell line assay without decreasing transcript expression of a Ghrelin O-acyltransferase on octanoylated ghrelin production. Ghrelin exerts orexigenic effect following octanoyl modification at serine 3 (Nakajima et al., 2018). Ghrelin acylation is catalyzed by (GOAT) using fatty acyl-coenzyme A as a substrate. The antiobesity action mediating octanoylated ghrelin production was attributed to the presence of carboxyl group.

## Asiatic acid in cerebral ischemia

Stroke or cerebral ischemia is one of the major cause of mortality and morbidity worldwide. The only available drug for acute management of stroke is tissue plasminogen activator (t-PA). Various AA containing plants showed neuroprotective in various *in vitro* and *in vivo* studies (Bonfill et al., 2006). Tabassum et al. (2013) studied the neuroprotective property of *C. asiatica* enriched in AA in a rat model of middle cerebral artery occlusion followed by reperfusion. The extract containing AA was found to improve neurobehavioral activity and diminish infarction volume along with the restoration of histology of brain mediated *via* its antioxidant activity. Krishnamurthy et al. (2009) for the first time showed that orally administered AA exerted neuroprotective effects during pre-ischemic and post-ischemic periods in a murine model of permanent cerebral ischemia. AA reduced infarct volume by 26 to 60% post-ischemia and improved neurological and behavioral deficits. Further, immunostaining was performed to determine IgG, a marker of blood-brain barrier integrity and cytochrome c, a marker of apoptosis and showed that AA treatment reduces blood-brain barrier permeability and mitochondrial injury. To elucidate the mechanism, HT-22 cells were exposed to oxygen-glucose deprivation and showed that AA improves cell viability and mitochondrial membrane potential.

In order to investigate the pharmaceutical development and clinical usage, a dose-response study including pharmacokinetics, safety, and efficacy was conducted employing multiple stroke models in animals. AA injected intravenously exhibited a half-life of 2.0 h and wide therapeutic margin of safety along with good efficacy. AA reduced infarct volume and improved the neurological outcome. Further, these effects replicated in female hypertensive rats as well. The authors showed that amelioration of mitochondrial dysfunction and matrix metalloproteinase-9 was the underlying mechanism of neuroprotection by AA (Lee et al., 2012). Although developing new therapeutic approaches for acute ischemic stroke has offered some successes, but failures have been more. Recent research has reported that free radical scavenging and anti-inflammatory neuroprotective strategies could be useful, with some conflicting data in animal models and humans concerning acute ischemic stroke. The combinations of neuroprotection and neurorecovery using the antioxidants of natural origin as well as modern drugs are ongoing for evaluation in experimental studies. Till date, the only therapy for acute stroke is tissue-plasminogen activator (t-PA). In a rat model of focal embolic stroke, AA and t-PA co-treatment reduced the infarct volume and diminished the release of cytochrome c and apoptosis-inducing factor from mitochondria of the ischemic brain (Lee et al., 2014). Furthermore, it showed neuroprotection against L-glutamate induced neurotoxicity in primary cortical neurons. The findings suggest that the combination of AA with t-PA is superior in improving neurological outcome and reducing infarct volume. In a recent study, AA was found to protect against intracerebral hemorrhage-induced secondary brain injury by mediating MAPKs signaling pathway as AA acts as a p38 MAPK agonist and may work in synergy with a P2X7 antagonist, BBG (Wen et al., 2017). Taken together, the results demonstrated many neuroprotective activities of AA to the ischemic brain. Integrating the excellent efficacy and safety profile as well as permeation to the brain, AA seems to be a promising candidate for clinical and pharmaceutical development in stroke treatment.

## Asiatic acid in hypertension

Hypertension is one of the leading common chronic diseases across the world and accounts for a large proportion of cardiovascular morbidity and mortality. It gives rise to numerous diseases including stroke, congestive heart failure, chronic renal failure, coronary artery diseases, ischemic heart disease, and peripheral arterial disease (Lewington et al., 2002). The redox-sensitive signaling pathways regulate vascular function therefore in regulating blood pressure and remodeling, targeting of oxidative stress, and inflammation plays an important therapeutic role. Pakdeechote et al. (2014) for the first time reported the antihypertensive effects of AA. The antihypertensive effect of AA in Nω-nitro-L-arginine methyl ester hydrochloride-induced hypertensive rats found associated with improved hemodynamic and vascular function alongwith reduced oxidative stress as evidenced by declined vascular O_2_^(.)^ generation, raised expression of NOx and eNOS along with downregulation of p47 (phox) expression. AA found to improve bioavailability of NO which regulates vascular tone by triggering soluble guanylyl cyclase (sGC) to produce cyclic guanosine monophosphate (cGMP) thus modulating contraction and relaxation of vascular smooth muscle cells (Pakdeechote et al., 2014).

In another study, Bunbupha et al. (2015) has demonstrated the effect of AA on cardiovascular remodeling and possible mechanisms involved in hypertensive rats induced by L-NAME. AA was found to reduce blood pressure, restore NOx, and eNOS/iNOS, dampen cardiovascular remodeling as well as inhibiting lipid peroxidation and TNF-α induction. In a recent study, AA attenuated renal hypertension by a multimodal mechanism including ACE inhibition to inhibit the AngII-AT_1_R and NADPH oxidase-NF-κB pathway (Maneesai et al., 2017). AA attenuated hemodynamic alterations (high blood pressure, heart rate, hind limb vascular resistance, and low hind limb blood flow), RAS activation (increased angiotensin II and angiotensin-converting enzyme activity in blood with enhanced AT_1_R and suppressed AT_2_R expression), oxidative stress (low levels of nitric oxide metabolites with increased generation of superoxide, MDA, and upregulation of gp91^phox^ protein expression), and inflammation (increased TNF-α, NF-κB, and p-NF-κB expressions) in renal hypertensive 2K-1C rats. The effects found comparable to captopril, an ACE inhibitor and suggest that AA has potential in alleviating hypertension. AA exhibited cytoprotective role by regulating lactate signaling cascade against lactate-induced cell death by reducing oxidative stress and the activation of mitochondria-dependent apoptosis along with enhanced mitochondrial monocarboxylate transporter 1 expression in neonatal rat cardiomyocytes (Gao et al., 2016).

In addition to achieve the recommended targets of blood pressure, the therapeutic approaches also consider the treatment of hypertension-related cardiovascular damage. AA was found to ameliorate myocardial ischemia/reperfusion in human myocardial cell lines (AC16) by inhibiting apoptosis and activating Akt/GSK-3β/HIF-1α pathway (Wu et al., 2017). AA inhibited hypoxia-induced cell death by regulating the miR-1290/HIF3A/HIF-1α axis as HIF-3α is a negative regulator of HIF-1α and mRNA is a potential target of miR-1290. The abrogation of the protective effect of AA following knockdown of miR-1290 demonstrated miR-1290 as a promising therapeutic target in myocardial ischemia-reperfusion injury. Further, AA showed cytoprotection in neonatal rat cardiomyocytes by reducing ROS production and inhibiting loss of Δψm as well as apoptosis (activated cytochrome c, cleaved caspase-9, and caspase-3 and MCT1) by regulating the lactate signaling cascade in mitochondria (Gao et al., 2016). The findings suggest that the therapeutic promise of AA in cardioprotection however future studies are needed for translation of effects from animals to humans.

## Asiatic acid in osteoporosis

Osteoporosis, a common disease in old age particularly in women following menopause often characterized by a low bone mass and deteriorated bone tissues (Pagliari et al., 2015). AA found to modulate bone marrow mesenchymal stromal cells differentiation and caused a lineage shift away from adipocytes following peroxisome proliferation-activated receptor-gamma (PPAR-γ) inhibition through C/EBPβ-independent mechanisms (Li et al., 2014). Bone marrow mesenchymal stromal cells are pluripotent cells that differentiate into adipocytes and osteoblasts involving transcription factors; PPARγ and Cbfa1/Runx2 that mediate adipogenesis (Lee and Ge, 2014) and osteogenesis, respectively (Ahdjoudj et al., 2004). C/EBPβ is a key upstream regulator of PPAR-γ, which is important for adipogenesis (Lehrke and Lazar, 2005). Considering the fact that a patient with diabetes mellitus is at a high risk of fracture depending on the duration of disease as well as the quality of metabolic control, AA appears a novel agent for further exploration in osteoporosis treatment.

## Asiatic acid in photoaging

Photoaging also known as premature skin aging, is characterized by a complex process of skin changes induced over time by solar radiation or ultraviolet light exposure as well as infrared (IR) and visible light which may also cause extrinsic skin aging. Matrix metalloproteinase families of enzymes play a significant role in extracellular matrix remodeling and collagen degradation. The enhanced activity of these enzymes following exposure to UV light results in premature aging of skin and superimposed on the changes caused by chronologic aging (Han et al., 2014). The popular approach to control photo damage involves inhibition of MMPs, activation of proteasome, pro-collagen, and collagen production along with uniform packaging of new collagen fibers. The current advancement in cosmetic sciences demonstrates that inhibition of MMP induction alleviates photoaging induced by UV as evidenced by the prevention of collagen degradation (Fisher et al., 2002). UVA-irradiated human skin generates ROS that results in oxidative damage to biomolecules such as DNA, proteins, and lipids (Hanson and Clegg, 2002). DNA damage causes activation of p53 that further leads to apoptotic cell death of keratinocytes and results in disruption of the epithelial structures (Qin et al., 2002). AA found to protect against skin aging caused by ultraviolet light, genetic factors and environmental pollution. AA modulated signaling pathways involved in UVA-photoaging, particularly their role on generation of ROS, stimulation of MMPs, and p53 expression in the HaCaT cells (Soo Lee et al., 2003). The effects were comparable to retinoic acid, a clinically available anti-wrinkle agent. AA blocked UVA-induced activation of MMP-2 and p53, ROS generation and consequent lipid peroxidation. AA (5 mM) also protected against UVA-induced ROS and MMPs formation in human skin keratinocyte cells; HaCaT (An et al., 2012). AA in HaCaT cells reduced glycative stress by blocking AGE-RAGE interaction, inhibiting MAPK pathway and MMP expression along with amelioration of oxidative and inflammatory stress. AA also exhibited potent free radical scavenging property and inhibited pro-inflammatory cytokines against LPS-induced inflammatory response in human corneal epithelial cells (Chen et al., 2017). It also inhibited LPS-induced p-Akt and TGF-β and retained glutathione levels. AA appears an effective agent to prevent UVA-induced photo aging by regulating signaling pathways in human skin cells. However, the cosmetic applications of AA in humans require more extensive future studies.

## Asiatic acid in parkinson's disease

Parkinson's disease is a chronic neurodegenerative movement disorder characterized by loss of dopamine-producing neuronal cells in the substantia nigra and aggregates of the protein alpha-synuclein (Thome et al., 2016). Various experimental studies has demonstrated that the effects and mechanism of AA in Parkinson's disease. Xiong et al. (2009) demonstrated AA protected against cellular injury and mitochondrial dysfunction induced by H_2_O_2_ or rotenone in SH-SY5Y cells. AA inhibited dissipation of mitochondrial membrane potential and rise in voltage-dependent anion channel induced by rotenone challenge (Xiong et al., 2009).

In a recent study, Nataraj et al. (2017) demonstrated the antioxidant, cytoprotective and antiapoptotic activities of AA against rotenone-induced cytotoxicity in SH-SY5Y cells. The *in vitro* results were further confirmed in 1-methyl-4-phenyl-1,2,3,6-tetrahydropyridine (MPTP)-induced Parkinson's disease in mice (Chao et al., 2016). AA found bioavailable in the striatum and reduced free radical generation along with raised glutathione levels. It also dose-dependently reduced nitric oxide, 3-nitrotyrosine, and prostaglandin E2, induction of pro-inflammatory cytokines and inflammatory mediators iNOS and COX-2. AA further reduced the expressions of bax, apoptosis-inducing factor, caspase-3, TLR2, NF-κBp65, p47(phox), and gp91(phox) and induced Bcl-2 expression in the striatum. The enhanced expression of tyrosine hydroxylase and decreased α-synuclein as well as TLR4 expression in the striatum indicated the amelioration of dopaminergic neurodegeneration, a pathologic hallmark of Parkinson's disease. It also raised the levels of dopamine in the striatum and enhanced brain-derived nerve growth factor and glial cell line-derived neurotrophic factors, which modulated various signaling cascades. The neurotrophic effect of AA was showed in mice model of PD induced by MPTP/probenecid (Nataraj et al., 2017). AA administration ameliorated motor dysfunction and reduced expressions of neurotrophic factors and tyrosine kinase receptors (TrkB) along with improved dopamine levels. AA further inhibited phosphorylation of MAPK/P38 proteins such as JNK and ERK and enhanced phosphorylation of PI3K, Akt, GSK-3β, and mTOR mediating PI3K/Akt/mTOR signaling pathway. This provides mechanistic evidence on the antiparkinsosnian activity of AA.

In another study, AA present in polar fractions of *C. asiatica* extract showed neurite elongation in the presence of nerve growth factor in human SH-SY5Y cells (Soumyanath et al., 2005). AA isolated from *C. asiatica* also showed potent induction in neurite outgrowth and neurofilament by promoting neuronal differentiation of PC12 cells (Jiang et al., 2016). The nerve differentiation property of AA mediating MEK signaling pathway appears a promising target to treat neurodegenerative diseases wherein neurotrophin deficiency plays an important role in etiopathogenesis. Recently, AA has been shown to attenuate neurotoxicity caused by a commonly abused drug, methamphetamine in dopaminergic cells viz. human neuroblastoma cells (SH-SY5Y), murine microglial cells (BV2) and primary culture of rat embryo mesencephalic neurons (Park et al., 2017). AA attenuated TNF-α and IL-6 secretion, TNFR expression, NF-κB/STAT3 translocation, and ERK phosphorylation along with proteolytic breakdown of caspase-3 and PARP. Additionally, it reduced the expression of bax and increased Bcl-xL expressions with stabilization of mitochondrial membrane. Taken together, all these beneficial effects including enhanced dopamine levels and neurogenesis are clearly suggestive of the therapeutic potential of AA in Parkinson's disease.

## Asiatic acid in pancreatitis

Acute pancreatitis represents a therapeutic enigma and leads to majority of the hospital admissions in gastrointestinal clinics. AA was found to attenuate pancreatitis in mice induced by cerulein (Xiao et al., 2017). AA was shown to inhibit mRNA expression of pro-inflammatory cytokines along with a reduction in levels of amylase and lipase in serum. AA also salvaged histology and reduced myeloperoxidase activity. Further, it also improved the viability of pancreatic acinar cells and attenuated NF-κB activity.

## Asiatic acid in respiratory diseases

AA showed effective in an animal model of chronic obstructive pulmonary disease by exerting protection against cigarette smoke-induced pulmonary inflammation (Lee et al., 2016). It reduced ROS generation, norepinephrine activity, pro-inflammatory cytokines, and infiltration of inflammatory cells in bronchoalveolar lavage fluid in mice. It also diminished overproduction of mucus, MCP-1 expression and MAPKs as well as NF-κB activation along with improved expression of HO-1 and SOD3 in lung tissues (Lee et al., 2016). AA was found to attenuate acute lung injury in rats induced by spinal cord injury. AA diminished pulmonary edema and infiltration of neutrophils, activation of myeloperoxidase, pro-inflammatory cytokines and attenuating oxidative stress mediated by enhancement of Nrf2 and attenuation of NLRP3 inflammasome favorably modulated histologic changes. Additionally, it also attenuated lung wet-to-dry weight ratio and pulmonary permeability index (Jiang et al., 2016b).

In another study, AA attenuated LPS-induced acute lung injury (Li and Xiao, 2016). AA treatment inhibited activation of TLR4, pro-inflammatory cytokines and NF-κB by inhibiting TLR4/NF-κB signaling pathway. It prevented the pathological changes of lungs as evidenced by the histological evidences and showed a reduction in myeloperoxidase activity and infiltration of neutrophils in bronchoalveolar lavage fluid (Li and Xiao, 2016). Recently, AA was found to protect bleomycin-induced pulmonary fibrosis, a life-threatening condition in humans on chemotherapy with bleomycin (Dong et al., 2017). AA inhibited inflammation, fibrosis, and lung injury by suppressing TGF-β1, collagen I, Collagen III, α-SMA, and TIMP-1 as well as Smad and ERK1/2 activation. These studies have revealed the therapeutic potential of AA for the treatment of acute lung injury.

## Asiatic acid in alzheimer's disease

Alzheimer's disease (AD), an age-related neurodegenerative disorder is characterized by severe loss of memory and impaired cognitive functions. Among multifactorial pathogenesis, oxidative stress and neuroinflammation contributed numerus events in triggering amyloid plaque formation. For the first time, Jew et al. (2000) synthesized a series of AA derivatives and screened their effect against neurotoxicity induced by Aβ. AA was found most effective (97%) and potent against Aβ neurotoxicity. The extract of *C. asiatica* is well-reputed in ancient medicine for neuroprotective properties and cognitive benefits. In numerous *in vitro* and *in vivo* studies, AA reported effective in AD and recently patented by the Hoechst Aktiengesellschaft for the treatment of dementia and an enhancer of cognition (patent Number: EP 0383171A2). AA also showed beneficial in preventing cognitive impairment subsequent to a reduction in oxidative stress induced by quinolinic acid in rats (Loganathan and Thayumanavan, 2018). AA showed to correct spatial memory deficit in novel object location test and attenuate oxidative stress by improving antioxidants and abating mitochondrial dysfunction manipulating oxidative phosphorylation.

Further, Xu et al. (2012) confirmed the cognition-enhancing effect in neonatal mice challenged with monosodium glutamate and underlying mechanism in human neuroblastoma SH-SY5Y cells. AA ameliorated neuronal damage, restored antioxidants, and attenuated lipid peroxidation concomitant to improved cognition in mice. In the quest for drug to modify the disease process of AD, Patil et al. (2010) showed that AA modulates numerous pathophysiological enzymatic pathways including BACE1, ADAM10, IDE, and NEP associated with the formation of amyloid-β (Patil et al., 2010). BACE1 as a rate-limiting enzyme regulates generation of amyloid-β from an amyloid-beta precursor protein (AβPP), whereas ADAM10 controls the non-amyloidogenic processing of AβPP in cortical neurons obtained from rat. The enzymes IDE and NEP are involved in effectively degrading Aβ. AA controls Aβ formation and degradation by reducing BACE1 and increasing the activities of IDE and ADAM10.

Zhang et al. (2012) showed that the neuroprotective effects of AA are mediated by lipid signaling pathways involving ceramides, biologically active lipids which are derived from sphingosine that play significant role in regulating mitochondrial function and subsequent neuronal cell death (Arboleda et al., 2009). There is increasing evidence suggesting that ceramides may be involved in several neurodegenerative disorders including AD (Jana et al., 2009). AA was dose-dependently reduced ceramide-induced cell death and loss of mitochondrial membrane potential. Further, AA also diminished ROS and cytosolic release of HtrA2/Omi, rise in the expressions of caspase 3, Bax, and dephosphorylation of ERK1/2. The neuroprotective activity of AA appears regulated by ERK1/2 signaling pathway that regulates oxidative stress and mitochondria-dependent apoptosis. The authors suggest the neuroprotective activity of AA and indicated that ceramide based cell culture model can be explored as a screening tool for evaluation of the neuroprotective potential of natural compounds (Zhang et al., 2012).

Aluminum is a widely distributed environmental toxicant that upon accumulation in the brain shows associated with neurodegenerative diseases, especially AD. Recently, AA has showed neuroprotective against aluminum maltolate-induced neurotoxicity (Ahmad Rather et al., 2018). AA found to enhance cell viability, ameliorate mitochondrial membrane dysfunction, and resultant oxidative stress and apoptosis-regulating AKT/GSK-3β signaling pathway in neuroblastoma cells (SH-SY5Y). Further, the authors in another study reconfirmed AA's neuroprotective effect against aluminum chloride-induced rat model of AD. AA mitigated rise in aluminum, activity of acetylcholinesterase and gamma secretases along with amyloid β_1−42_, glial fibrillary acidic protein and ionized calcium binding adaptor molecule-1. AA also reduced inflammatory mediators iNOS, COX-2, and NF-κB in the hippocampus and cortex (Ahmad Rather et al., 2018).

In a study, the authors examined numerous derivatives of asiaticoside against for Aβ-induced neurotoxicity in B103 cells and concluded that AA exhibited potent inhibition of free radical production and apoptotic cell death (Mook-Jung et al., 1999). The derivatives also inhibited H_2_O_2_-induced cytotoxicity with most potent activity by AA. Though, in hippocampal slices, AA actions on synaptic transmission, paired-pulse facilitation, or induction of long-term potentiation in CA1 field were found independent of did not alter n-Methyl-D-aspartic acid (NMDA) receptors. This is suggestive of no physiological change at hippocampal level with the therapeutic concentration. However, this aspect should be investigated *in vivo*. AA was found to increase hippocampal neurogenesis augmented double cortin in the hippocampus and Notch1 protein levels, a novel neuron marker in the neural stem cells hippocampus (Sirichoat et al., 2015). Further, AA reduced brain aging in the D-galactose-inducedageing brain in mice (Chao et al., 2015). AA improved antioxidants, NAPDH oxidase expression, inhibited inflammation and reduced apoptosis reflected by reduced Bax, cleaved caspase-3 expressions, and maintained Bcl-2 expression. AA also reduced RAGE production, AGE and carboxymethyl lysine expression along with p-p38, p-JNK, and CD11b expressions. The preserved expression of brain-derived neurotropic factor and increased glial fibrillary acidic protein demonstrate the neuroprotective effects in aging brain.

Lee et al. (2000) synthesized 36 derivatives of AA which exerted neuroprotective effect by suppressing cytotoxicity in rat cortical neurons. The derivatives were found to be more potent than AA and raised glutathione, reduced NO production and enhanced antioxidants defense mechanisms (Lee et al., 2000). Kim et al. (2004) synthesized 36 derivatives of AA and evaluated their effect on cognition in scopolamine-induced memory impairment using water maze, passive, and active avoidance tests. These derivatives were found to improve cognition without affecting the learning process and improved cholineacetyltransferase activity in a cholinergic neuroblastoma cells (S-20Y) demonstrated a mechanism for improving memory in dementia.

The clinical use of a chemotherapeutic drug, 5-fluorouracil is often compromised due to potential cognitive impairments in patients. AA corrected impairment in spatial working memory and neurogenesis in rats receiving 5-FU (Chaisawang et al., 2017). AA improved spatial memory in novel object location test subsequent to a reduction in proliferation and survival of cells in the subgranular zone of the hippocampal dentate gyrus (Chaisawang et al., 2017). The studies are suggestive of the protective effects of AA on chemotherapy-associated adverse effects. Taken together, all these studies suggested AA a novel agent for pharmaceutical development in the management of AD.

## Asiatic acid in periodontitis

Periodontitis occurs following chronic accumulation of bacterial plaque due to the regulated response to bacterial infection directed by inflammatory cells of the host immune system. AA found to inhibit pro-inflammatory cytokines and improve cell viability in lipopolysaccharide (LPS)-challenged HGFs or RAW264.7 cells (Hao et al., 2017). In cells, AA reduced LPS-challenged p65 NF-κB phosphorylation and led to the abrogation of preventive effects. AA shows promising potential for the treatment of periodontitis. Further, AA exhibited an antifibrotic effect in primary human buccal fibroblasts induced by arecoline (Adtani et al., 2017). AA showed salvage of fibroblast morphology mediating inhibition of transforming growth factor-β1, collagen 1 type 2, and collagen 3.

## Asiatic acid in keloid

Keloids are benign dermal scars that occur because of abnormal wound healing. They are characterized by the overt generation of extracellular matrix leading collagen deposition due to exuberant fibroblast dynamics. The high rate of recurrence after surgical procedures and scarcity of effective treatment necessitate development of new treatment strategies (Gauglitz et al., 2011). In one of the recent studies, the glycoside of AA suppressed collagen deposition in keloid fibroblasts (Tang B. et al., 2011). Furthermore, AA also suppressed expression of plasminogen activator inhibitor-1 (PAI-1) and collagen in TGF-β1-induced keloid fibroblasts by activating Peroxisome proliferator-activated receptor gamma (PPAR-γ) (Bian et al., 2013). In earlier studies, PPAR-γ activation showed to inhibit TGF-β/Smad signaling and a resultant decrease in collagen deposition and epithelial-mesenchymal transition in fibrosis of different organ tissues (Wang et al., 2007; Ferguson et al., 2009). AA thus appears to be a promising future agent for the management of keloids.

## Asiatic acid in wound healing

*C. asiatica* in Asian countries are in use since long time in ancient and folk medicine due to its potential in boosting memory, improving skin conditions in leprosy and psoriasis and aiding in to wound healing (Cheng and Koo, 2000). Wound healing is a complicated process; there are nearly three overlapping phases, inflammation, granulation, and remodeling (Gurtner et al., 2008). The value of *C. asiatica* extracts in the healing process of small wounds, hypertrophic scars and burns have been recently reviewed (Bylka et al., 2014). Alpha Centella cream containing an extract of *Bulbine frutescens* and terpenoids (AA, madecassic acid, and asiaticoside) from the plant *C. asiatica* is attributed to promote wound healing. The effects such as hydrating, antibacterial, anti-inflammatory, and antimyofibroblast production and promotion of maturation of the scar following the synthesis of type I collagen, which assist in scar development along with modern aesthetic surgical techniques (Widgerow et al., 2000).

Titrated extract of *C. asiatica* containing asiaticoside, AA and madecassic acid demonstrated effective in scar management, systemic scleroderma and keloids (Hong et al., 2005). Triterpenic derivatives were showed to stimulate collagen synthesis (Laugel et al., 1998). The emulsion type formulations protect triterpene ingredients from oxidation and modulate their release upon topical application. Numerous extract fractions of *C. asiatica* such as hexane, ethyl acetate, and methanol reported to contain AA and showed to promote wound healing in rat models of incision and burn wound (Somboonwong et al., 2012). AA also reduced tensile strength of incision wound along with developed epithelialization and keratinization. AA alone as well as other triterpenoids of *C. asiatica* showed to promote collagen I synthesis without contribution of the sugar moiety of the molecule for this biological activity of asiaticoside and AA (Bonte et al., 1994).

A formulation of *C. asiatica* known as TECA and its constituent AA showed to enhance hydroproline, glycosaminoglycan synthesis, total protein, DNA, dry weight, collagen, and uronic acid in the wounds as assessed in wound chamber model wherein stainless steel wound chambers were surgically implated beneath the skin of rats (Maquart et al., 1999). In another study in primary human skin fibroblasts originated from healthy human foreskin samples, AA found to promote burn wound healing (Wu et al., 2012). Further, in mice AA administered orally stimulated proliferation of cells, promoted synthesis of collagen, and restored MMP-1/TIMP-1 balance by regulating TGF-β/Smad signaling pathway (Wu et al., 2012). Using gene microarrays and real-time reverse transcription polymerase chain reaction, the effect of AA on the expression of 1053 human genes in human fibroblasts were assessed (Coldren et al., 2003). AA found to activate growth factor genes and genes involved in angiogenesis and remodeling of extracellular matrix. The findings validated the claims of extract in wound healing in traditional medicine and scientifically prompt use of AA for the treatment of wound healing and microangiopathy.

Many synthetic derivatives of AA were developed and assessed for the wound healing effects using a tensile strength assay and a wound area assay (Jeong, 2006). Among the 10 synthesized derivatives, ethoxymethyl 2-oxo-3,23-isopropylidene-asiatate was found most potent, rapid-acting and most efficacious in wound healing activity. AS 2-006A, a derivative of AA was developed as a new wound healing agent and its pharmacokinetics was established (Kim et al., 1999). Yuan et al. (2013) further showed the protective effects and mechanisms of AA against oxygen-glucose deprivation/re-oxygenation (OGD/R) injury in PC12 cells, Na_2_S_2_O_4_ combined with low glucose-induced damage of PC12 cells. MTT method was used to evaluate cell survival. Ultraviolet spectrophotometry was performed to determine lactate dehydrogenase (LDH) leakage, lactic acid (LD) content, intracellular superoxide dismutase (SOD), malondialdehyde (MDA), and cellular Caspase-3 activity. Flow cytometry was applied to assay cell apoptosis. Na2S2O4 combined with low glucose increased cell survival rate significantly. Increase in cell survival rate, alleviation of LDH leakage, inhibition of LD production, promotion of SOD activity, reduction in MDA content, decreased cell apoptosis, and inhibition of caspase-3 activity observed when cells were pre-treated with AA. An evident protective effect of AA against OGD/R injury in PC12 cells was observed and the possible mechanisms identified were elimination of free radicals and inhibition of cell apoptosis.

## Asiatic acid in liver diseases

AA in many studies revealed as a potent hepatoprotective agent by attenuating mitochondrial stress and improving antioxidant defense mechanism (Lee et al., 2009; Ma et al., 2009). For the first time, in a preliminary study AA showed hepatoprotective against CCl4, acetaminophen and cadmium chloride induced liver injury in male CF-1 mice (Liu et al., 1994). AA isolated from *Terminalia catappa* L. showed a dose-dependent free radical scavenging activity against carbon tetrachloride-induced acute liver damage and D-galactosamine (D-GalN)-induced hepatocyte injury (Gao et al., 2004).

In another study, AA protected against LPS and D-GalN-induced liver injury in mice. AA attenuated mitochondrial proliferation, improved nuclear condensation and restored liver enzymes. It modulated mitochondrial permeability transition induced by Ca^2+^ in addition to inhibiting mitochondrial swelling, membrane potential dissipation and release of matrix Ca^2+^ in liver mitochondria and reducing voltage-dependent anion channels and blocked translocation of cytochrome c from mitochondria to cytosol (Gao et al., 2006). The structural modification of AA at C2 functional group provided compounds with improved hepatoprotective effects in comparison to silymarin (Jeong et al., 2007). The derivatives of AA such as 3beta, 23-dihydroxyurs-2-oxo-12-ene-28-oic acid and 3beta, 23-dihydroxyurs-12-ene-28-oic acid attenuated hepatotoxicity in cultures of rat hepatocytes induced by carbon tetrachloride (Lee et al., 2009). These derivatives inhibited lipid peroxidation and maintained endogenous antioxidants regulating cellular antioxidant defense system. Wei et al. (2013) showed hepatoprotective effect of AA isolated from *Potentilla Chinensis* against ethanol-induced liver injury as evidenced by reduced liver enzymes in serum and histopathological salvage. AA elicits hepatoprotective effects against D-GalN or CCl4 –induced injury in rat hepatocytes (Ma et al., 2007). AA enhanced viability of the hepatocyte, ameliorated oxidative stress and loss of mitochondrial membrane potential (Ma et al., 2007).

Further, the hepatoprotective mechanism of AA was demonstrated in D-GalN/LPS-induced hepatotoxicity in hepatocytes and kupffer cells. AA corrected the impaired cell redox, reduced LTC4 expression by regulating extracellular signal-regulated kinase 1/2 (ERK 1/2) and NF-κB pathway (Ma et al., 2009). AA also ameliorated fulminant hepatitis induced by concanavalin A-activated T cells involving mitochondrial pathways (Guo W. et al., 2012). AA restored liver enzymes, and regulated Fas-mediated death-receptor apoptotic pathway by inducing apoptosis of activated CD4(+) T cells.

Furthermore, AA also provided hepatoprotective effects against hepatic steatosis induced by high-fat-diet-induced in mice (Yan et al., 2014a). AA reduced body weight, epididymal fat and triglyceride levels in liver and plasma along with food and water intake. AA reduced expression of enzymes, acetyl coenzyme A carboxylase, fatty acid synthase, stearoyl CoA desaturase, and 3-hydroxy-3-methylglutaryl coenzyme A reductase in mice. A significant reduction in oxidative stress, induction of pro-inflammatory cytokines, inflammatory infiltrate, and lipid accumulation was observed in liver. Further, AA reduced plasma insulin secretion and HOMR-IR as well as diminished NF-κBp50, NF-κBp65, p-p38, and p-JNK expression in liver. In another model of cyclophosphamide-induced hepatotoxicity and immunosuppression in rats too, AA protected against the immune-mediated liver diseases by improving hematology, liver function markers, antioxidants and inhibiting lipid peroxidation, and pro-inflammatory cytokines as well as preserving liver, intestine, and spleen histopathology and weights of spleen and thymus (Duggina et al., 2015).

Another major concern in liver diseases are liver failure which mainly involves liver fibrosis that lacks successful treatment. The liver fibrosis, which eventually results in cirrhosis, arises by over-accumulation of fibrous components in the extracellular matrix in liver. TGF-β1 is a vital mediator in the process of liver fibrosis (Meindl-Beinker and Dooley, 2008). On the other hand, overexpression of Smad7 is capable of preventing TGF-beta1 and angiotensin II-induced fibrosis *in vitro* (Yang et al., 2009). AA attenuated liver fibrosis *in vitro* by preventing collagen matrix generation and autocrine effect of TGF-β1 (Tang et al., 2012). AA *in vivo* also ameliorated liver fibrosis in rats induced by carbon tetrachloride and TGF-β1 activated rat hepatic stellate cell line (HSC-T6) *in vitro* (Tang et al., 2012). AA fibrogenesis in HSC induced by TGF-β1 as evidenced by induction of Smad7 expression, inhibition of Smad2/3 activation, myofibroblast transformation, HSC activation and alpha-smooth muscle actin as well as collagen matrix. Further, in order to develop antifibrotic agents for liver, novel derivatives of AA were synthesized following strucutural modification and elicit potential antifibrotic action against CCl4-induced liver injury (Li et al., 2015).

In another study, AA also showed hepatoprotective in Kupffer cells or RAW264.7 cells challenged with LPS/H_2_O_2_ and mice underwent hepatic ischemia/reperfusion, which leads to perioperative morbidity and mortality (Xu et al., 2017). AA ameliorated liver injury by countering oxidative stress, inflammation, and apoptotic cell death along with NLRP3 inflammasome activation. AA inhibited LPS/H_2_O_2_-induced NLRP3 inflammasome activation in kupffer cells and RAW264.7 cells. Abrogation of the protective effects of AA by GW9662, a PPARγ demonstrated that the protective effects were mediated by PPARγ activation. Thus, activation of PPARγ/NLRP3 inflammasome signaling pathway including JNK, p38 MAPK, and IκBα appear hepatoprotective.

## Asiatic acid as a spermicidal agent

Spermicidal agents are dispersed and retained in the vagina to provide contraception. AA isolated from *Shorea robusta* showed spermicidal and antimicrobial activity in Sander-Cramer test and disc diffusion and broth dilution tests using human isolates of bacteria (*Escherichia coli;* ATCC25938 and *Pseudomonas aeruginosa 71*) and fungus (*Candida tropicalis*) (Bharitkar et al., 2013). The MIC of AA to immobilize spermatozoa was 125 mcg/ml and a moderate antimicrobial activity found for vaginal pathogens with minimal effect on normal constituent vaginal flora. The disruption of sperm plasma membrane appears to be the possible mechanism underlying the microbicidal as well as spermicidal action. The results suggest that AA has potential for its application as a microbicidal spermicide.

## Asiatic acid in malaria

AA found to elicit chemoprophylactic effects by diminishing parasitemia and anemia associated with murine malaria in rats infected with *Plasmodium berghei* (Mavondo et al., 2016a). AA treated rats showed better control of parasitemia than chloroquine, a standard drug for malaria. In another report, the same authors showed that AA pretreatment in rats before *Plasmodium berghei* infection restored food and water intake and enhanced percentage weight gain with reduced parasitemia whereas orally administered chloroquine failed to influence malaria induction (Mavondo et al., 2016b). Altered glucose homeostasis is a part of malaria. The drugs used in malaria treatment also cause hypoglycemia. AA has been shown to improve oral glucose response as well as food and water intake in addition to reduced parasitemia and lactate concentration in the *Plasmodium berghei* infected Sprague Dawley rats (Alfred et al., 2016).

The authors further showed the antimalarial effect of transdermal formulation of AA applied as on the shaven dorsal neck region of Sprague Dawley rats infected with *Plasmodium berghei*. Rats showed maintained body weight, food, and water intake concomitant to reduced parasitemia and the effects were comparable to chloroquine pectin patch. AA has been suggested to be a novel agent for malaria treatment as well as an adjuvant in countering hypoglycemia, which occurs during malaria or synergizes with the action of antimalarial drugs.

## Asiatic acid as food preservative

AA appears an effective food preservative and provides protection against bacterial contamination as well used as a bactericide in farms and slaughterhouses to promote environmental sanitation. 8 mg of AA in 100 g ground beef that is considered equal to 8 ppm showed potent anti-bacterial effects by increasing K^+^ release from the cytoplasm and/or mitochondria that leads to membrane damage and leaching out of nucleotides such as DNA and RNA from bacteria (Liu et al., 2015). Being natural, safe, tasteless, and odorless, AA was suggested to be used as a food flavor (Liu et al., 2015). The steamed and metal-chlorophylls complexation of the extracts from *C. asiatica* leaves was found better than untreated leaves and synthetic colorant. The extract is used as a yellow-green color in beverage and food and is also safe against Vero cells. AA was found effective against foodborne bacterial pathogens such as *Escherichia coli* (O157:H7), *Salmonella Typhimurium (*DT104), *Pseudomonas aeruginosa, Listeria monocytogenes, Staphylococcus aureus, Enterococcus faecalis*, and *Bacillus cereus* with minimal inhibitory and bactericidal concentrations in the range of 20–40 and 32–52 μg/mL in 6 h (Liu et al., 2015). AA also enhanced bacterial nucleotide leakage and attenuated growth of test bacteria in ground beef and the antibacterial effect was not affected by heat. AA has been suggested to be used as food additive and preservative to prevent contamination.

## Asiatic acid as antibacterial

AA has been shown to suppress growth, cell morphology, virulence factors, and biofilm formation by *Enterococcus faecalis* strains. It showed potent anti-biofilm activity without affecting hydrophobicity of bacteria and reduced survival as well as virulence (Wojnicz et al., 2017). AA isolated from *Melastoma malabathricum* L. showed antibacterial activity against numerous microbes in agar diffusion method (Wong et al., 2012). AA isolated from *Symplocos lancifolia* showed antibacterial against *Pseudomonas aeruginosa, Staphylococcus aureus, Enterococcus faecalis, Escherichia coli*, and several Gram-positive bacteria (Acebey-Castellon et al., 2011).

AA examined on silicone catheters and polystyrene microliter plates was found to inhibit biofilm formation in uropathogenic *Escherichia coli*. AA synergistically enhances the bactericidal activity of ciprofloxacin, a fluoroquinolone antibiotic used in the treatment of recurrent urinary tract infections caused by *Escherichia coli* (Wojnicz et al., 2015). In urinary tract infections, adhesion of the bacteria to the epithelial tissue and further colonization is a vital step. AA favorably influenced morphology, hydrophobicity and adhesion of uropathogenic *Escherichia coli* strain to the epithelial cells to reduce antibiotic resistance and thus reduced chances of recurrent infections (Dorota et al., 2013). AA also exhibited anti-biofilm activity on *Pseudomonas aeruginosa* biofilms grown in rotating disk reactors in combination with tobramycin and ciprofloxacin (Garo et al., 2007). AA isolated from leaf extracts of *Syzygium guineense* showed antibacterial activity against numerous microbes including *Escherichia coli, Bacillus subtilis*, and *Shigella sonnei* (Djoukeng et al., 2005).

## Asiatic acid as antifungal

AA and the plants containing AA have been shown effective in microbial infections of animals and plants along with an activity against fungi. AA isolated from stem extract of *Schisandra Grandiflora* showed cytotoxicity against human cancer cell line (HepG2) and antifungal activity on plant pathogens, *Alternaria alternata* and *Alternaria solani* (Shi et al., 2016). AA isolated from *Combretum laxum* showed an *in vitro* antifungal activity against *Candida albicans, Candidakrusei*, and *Cryptococcus neoformans* (Bisoli et al., 2008). Masoko et al. (2008) further demonstrated that the bioassay-guided fractionation of leaf extracts of *Combretum nelsonii* leaf extracts showed antifungal activity against fungal animal pathogens *Candida albicans, Cryptococcus neoformans, Aspergillus fumigates*, and *Microsporum canis* and *Sporothrix schenckii* (MIC = 0.2–1.6 mg/mL) with maximal potency against *Microsporum canis* and *S. schenckii*. AA in combination with another terpene compound; arjunolic acid showed cytotoxic on Vero monkey kidney cells. Further, in another study, Masoko et al. (2010) showed that AA and arjunolic acid elicited antifungal activity against *Candida albicans, Cryptococcus neoformans, Microsporum canis*, and *Sporothrix schenckii* when tpopicaly applied on the wounds of immunocompromised Wistar rats (Masoko et al., 2010). This antifungal activity was found comparable to amphotericin B when assessed by wound healing processes such as erythema, exudate, crust formation, swelling, and ulceration. Studies suggest that AA or the plants containing AA may be beneficial in fungal infections.

## Asiatic acid as antiviral

AA showed antiviral activity against enterovirus 71 (EV71), thus could be further exploited as antiviral candidates for EV71 infections (Zhao et al., 2014).

## Toxicity and safety of asiatic acid

The preclinical safety and toxicity testing are vital for evaluating the new molecules for the first time and in their further progression to different phases of clinical drug development. Regulatory toxicology is necessary for drug development, as preclinical toxicity has been considered a major barrier in the clinical development of a drug accounting for 59% failure at the preclinical phase (Waring et al., 2015). However, there are many studies that have been conducted in acute or chronic settings to demonstrate efficacy, but they are only indicative of relative safety and cannot ensure complete safety. A number of studies are available on the extract of *C. asiatica* containing AA and other major ingredients for safety and toxicity. However, there is no information available on AA as an individual agent in safety and toxicity assessments. The safety and toxicity of AA is based on the evidence arisen from toxicity studies with the extract only. Therefore, following efficacy evidence, regulatory toxicology data is essential to evaluate the drug in humans.

In one of the report, an analysis on drug development showed that out of 808 drug candidates developed by four major pharmaceutical companies during the period from 2000 to 2010, 356 compounds (44%) did not success to progress into clinical studies because of their potential toxicity. There are many human studies available to demonstrate the efficacy and safety of an extract of *C. asiatica*, which validates the traditional claims of the herb in therapeutics but none available for AA alone. Few human studies demonstrate the pharmacokinetics of an extract of *C. asiatica* and suggest its relative safety safe in humansat the tested doses (Anukunwithaya et al., 2017). But there is no clinical study wherein the safety and toxicity of AA have been reported as it has not been tested for its efficacy in humans yet. In animal studies, the doses studied for pharmacological effects are in the dose range of 20 to 500 mg/kg. In majority of the preclinical studies studies, AA was administered orally or intraperitoneally and was found pharmacological effective.

AA found protective against many pharmaceutical agents, pharmacological challenge agents, metals, environmental toxicants and industrial chemicals and was found to protect liver, a major site of drug metabolism. The extract of *C. asiatica* and active constituents including AA inhibit CYP2C9, CYP2D6, and CYP3A4 activities with most potent inhibition of CYP2C9 by AA that shows a potential risk for drug interaction. In an acute oral toxicity study in rats, the leaf extract of *Centella asiastica* showed LD_50_ of 200 mg/kg. The extract of *C. asiatica* showed a weak sensitizing action in skin irritancy test in guinea pigs and cause contact dermatitis (Hausen, 1993; Gomes et al., 2010). Madecassol, a brand containing the extract of *C. asiatica* that is available for prevention of cicatrization and as a wound healer also reported to cause contact dermatitis (Eun and Lee, 1985). The leaf extract of *C. asiatica* showed antispermatogenic and antifertility effects and was found to be non-genotoxic and non-carcinogenic.

Many preclinical studies showed the organo-protective effects of AA where AA was devoid of general, behavioral and systemic toxicity. In future, systematic regulatory toxicology studies are warranted to determine the safety parameters and further translate the beneficial effects in humans. The LD_50_ and no observed adverse effect level (NOAEL) should be investigated with different route of administration in acute and chronic settings in the most appropriate animal species following the US FDA guidelines. The preclinical data including dose-response relationship, pharmacokinetics, and regulatory toxicity data are vital to determine the first dose size in humans and clinical drug development of AA.

## Concluding remarks and future perspectives

This review highlights the pharmacological actions, molecular mechanisms, therapeutic potential, physicochemical properties, pharmacokinetics, drug delivery, and structural modifications of AA. The chemical scaffold of AA provides a platform for the synthesis of congeners and analogs of AA and the novel approaches of drug delivery improved pharmacokinetics and bioavailability of AA.

Integrating the evidences, it is apparent that AA has high potential to be developed for wound healing, neurodegenerative diseases and cancer. The main anticancer mechanisms of action of AA include inhibition of proliferation, angiogenesis, metastases, migration, tumourigenesis along with induction of apoptosis and activation of carcinogen-metabolizing enzymes. However, more *in vivo* studies still needed to confirm *in vitro* findings and reconfirm the reported findings with pharmacological and molecular mechanism. In addition, there is a contradiction between *in vitro* concentration and *in vivo* dose in certain types of cancers. Therefore, the pharmacokinetic studies with different routes of drug administration and more systematic *in vivo* studies needed to proceed in the direction of drug development and interpret the inconsistency between *in vitro* and *in vivo* observations. The currently available evidences are only focussing on efficacy parameters without almost negligible determination of pharmacokinetics and safety, which is impeding the search of novel therapeutics. Well-designed systematic determination of pharmacokinetics and long-term safety and toxicity studies following standard guidelines of regulatory toxicology need to be conducted in different types of animal models including non-rodent species to support the safety of AA and accelerate its pharmaceutical development.

Recently, antioxidants of natural origin tested in animal models revealed efficacious but there is still an ambiguity on the translation of benefits observed in experimental animals to humans. For a fair translational approach, it is imperative to conduct more and more studies with a concentrated focus on pharmacological basis of therapeutics rather than only antioxidant and anti-inflammatory approach of their basis in therapeutic benefits. Similarly, the screening of phytochemicals or plant extracts in cell lines also lacks clinical applicability due to physiologic and biochemical relevance. Therefore, *in vivo* studies are vital to reconfirm the *in vitro* findings.

In recent years, dietary interventions are one of the emerging trends in therapeutics to curtail chronic pathophysiological changes in chronic degenerative diseases. However, despite availability of preclinical data, more data needed to demonstrate and establish the use of AA as a nutraceutical or functional food in many diseases with a scientific basis. Taken together, the polypharmacological properties and molecular mechanisms, AA signifies its promise as an important pleiotropic multitargeted drug candidate of natural origin.

## Author contributions

All the authors provided important intellectual content, reviewed the content and approved the final version for the manuscript. Conceptualized the idea and revised manuscript: SO, SG, and CP. Proof read and extensive editing: SO, SG, and CS. Performed the literature search: MN, SG, KS, and SO. First draft of the manuscript: MN and SG.

### Conflict of interest statement

The authors declare that the research was conducted in the absence of any commercial or financial relationships that could be construed as a potential conflict of interest. The reviewer FC and handling editor declared their shared affiliation.
